# Organic–Inorganic Hybrid Nanofiber Membranes by Electrospinning: Engineering Features and Cytocompatibility

**DOI:** 10.1021/acsapm.5c01072

**Published:** 2025-07-11

**Authors:** Soraia A. R. Coelho, Liliana Grenho, Maria Helena Raposo Fernandes, Maria Helena Vaz Fernandes, José Carlos Almeida

**Affiliations:** † CICECO—Materials Institute of Aveiro, Department of Materials and Ceramic Engineering, University of Aveiro, 3810-193 Aveiro, Portugal; ‡ LAQV/REQUIMTE, Faculty of Dental Medicine, 26706 University of Porto, Rua Dr. Manuel Pereira da Silva, 4200-393 Porto, Portugal

**Keywords:** organic−inorganic hybrid membranes, poly caprolactone, electrospinning, *in situ* sol−gel, green route, physical-mechanical properties

## Abstract

Organic–inorganic (O/I) hybrid fibrous scaffolds represent a cutting-edge attempt for replicating the extracellular matrix (ECM) in tissue engineering. This study introduces an O/I hybrid composed of borosilicate-calcium elements incorporated with poly­(ε-caprolactone) (PCL) using an eco-friendly, *in situ* sol–gel process. The purpose of this research was to electrospin this hybrid composition into nanofibrous mats and investigate the impact of the electrospinning parameters on membrane properties. Critical electrospinning parameters (distance, flow rate, and voltage) were optimized to refine spinning behavior and assess their influence on fiber’s morphology and diameter, being optimal conditions were achieved with a distance of 12 cm, a flow rate between 125 and 150 μL·h^–1^, and a voltage in the range of 14–17 kV. Based on these conditions, two scaffolds with different fiber diameters (∼65 and 116 nm) were fabricated and compared. A homogeneous distribution of the inorganic phase inside the PCL matrix was successfully achieved, and the presence of hydrogen-bonding interactions between the polymer and the silica network was confirmed. Mechanical properties were improved by the introduction of borosilicate compositions, while the scaffold with the thinnest fibers presented the higher Young’s modulus and tensile strength. Contrary to polymer membrane control, the fibrous O/I hybrid showed a hydrophilic character, with distinct wetting properties depending on fiber diameter. *In vitro* acellular studies proved superior degradability and bioactivity of the hybrids, confirmed by surface apatite formation, compared with the neat PCL membrane. Higher cell proliferation was exhibited by the hybrids and the best cytocompatibility was favored by the lower fiber diameter. This sustainable, interdisciplinary approach to material design strengthens the development of green, next-generation materials with potential for bionanohybrid applications, particularly for tissue engineering.

## Graphical abstract

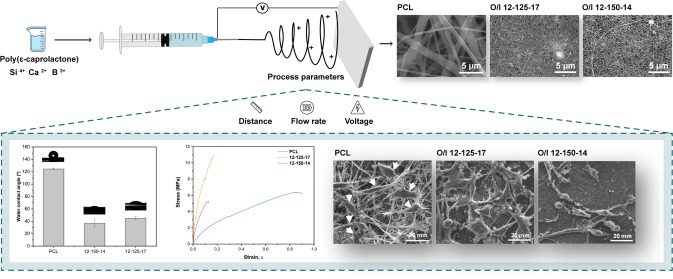

## 1 Introduction

Electrospinning (ES) is a simple, reproducible, and cost-effective process that enables the production of fibers of nano- and microscale diameters through the application of electrostatic forces.^1,2^ The fibers can be acquired with different morphologies, structures, and compositions depending on the specific application. Electrospun fibers have found extensive use in various sectors: wound dressing, tissue engineering, and filtration.^1,3^ In tissue engineering, the scaffold must simulate the extracellular matrix, and the electrospun fibrous scaffolds produced have shown to promote cell’s activity, due to the large surface area and porosity of the structures.^4^ Thus, the biological performance of scaffolds can be influenced by membrane morphology and microstructure, such as fiber diameter, pore size, porosity, and surface area.^4^ The biodegradability and biocompatibility of poly­(ε-caprolactone) make it widely used in tissue regeneration. Its superior rheological and viscoelastic properties make it an excellent candidate for fabricating fibrous structures through electrospinning. However, due to its inherent hydrophobicity and limited bioactivity, many studies have focused on incorporating bioactive components, such as bioactive glass (BG) nanoparticles, as fillers to produce composite nanofibers.^5,6^ Despite that a miscible solution is more rapidly achieved, this traditional composite production method can have its drawbacks. For example, particle agglomeration within the fibrous matrix, along with the insufficient interactions between the organic and inorganic parts, can potentially compromise mechanical properties and cellular responses. Organic–inorganic hybrids present a promising alternative, as they consist of organic and inorganic networks that are combined at the molecular level, resulting in a single phase at the nanoscale.^7^ Unlike conventional nanocomposites, hybrids are fabricated using a bottom-up approach, often encompassing sol–gel chemistry. Sol–gel facilitates not only a more homogeneous distribution of the inorganic component but also the use of mild conditions to not degrade the polymer. O/I hybrids can be classified into two classes, depending on the type of interaction that exists at the hybrid interface: class I, materials formed with weak interactions (van der Waals, hydrogen bond, *etc.*) and class II, materials with covalent bonds between phases.^7^ The principal advantage of O/I hybrids in comparison to nanocomposites is found in the sol–gel process, since the colloidal nature of the sol allows a uniform distribution of inorganic particles, typically only a few nanometers wide, thereby guaranteeing no phase heterogeneity at the nanoscale.^7^ The organic and inorganic interlink creates a set of distinct properties driven by their synergistic interactions.^7^ The synthesis of BG by sol–gel begins with a silicate precursor, for example, tetraethyl orthosilicate (TEOS). Using an acidic catalyst, TEOS undergoes hydrolysis and subsequently polymerizes into nanoparticles that gradually merge and cross-link, producing a gel. Before full gelation occurs, a polymer can be added during the condensation process, forming a hybrid.^7^ These hybrids can be further processed into fibrous structures *via* electrospinning. Pure silicate network alone often lacks sufficient bioactivity, but this can be overcome by adding therapeutic agents to its composition. Calcium (Ca) and boron (B) ions are recognized for their roles in bone metabolism, wound healing, growth factor stimulation, as well as angiogenesis activity.^8,9^ The combination of silicon, calcium, and boron elements as part of the inorganic phase in hybrids can have promising results in tissue engineering.

Designing a green solvent system that is compatible with both inorganic and polymeric solutions can be difficult. Until now, different solvents have been used to dissolve PCL, particularly toxic solvents, for instance, chloroform, dimethylformamide, and dichloromethane, since they can lead to beadless fibers.^10,11^ However, these solvents pose risks to the environment as well as to the operators and end users. Recent efforts have focused on finding safer solvents to reduce the risk, and they have been called “green” or “benign” solvents. For instance, acetone, formic acid, and acetic acid have yielded promising results in obtaining successful nanoscale fibers.^10,12,13^ Work has been published using these benign solvents in the fabrication of composite electrospun membranes based on PCL and bioactive glass (BG) particles.^5,14^ However, these approaches usually involve multistep and time-consuming procedures to incorporate presynthesized BG into the PCL solution. Generally, the BG particles are first produced by the sol–gel method and subsequently submitted to a high-temperature treatment and milling process. Although green solvent systems have been applied in PCL–BG composites, the direct combination of organic and inorganic solutions, produced *via* the sol–gel method, and using green solvents, remains unreported. Until now, only toxic solvents have been used to dissolve the polymer in O/I hybrids produced by *in situ* sol–gel.^11,15^ Electrospinning directly from a hybrid sol, achieved *via* the sol–gel process, offers several advantages, including reduced process steps and consequently lower production time, lower energy consumption, and greater homogeneity at the nanoscale. The introduction of borosilicate-calcium compositions through this *in situ* and green approach has been previously attempted by the present authors.^9^ However, in the present study, a distinct experimental protocol was developed using a different calcium precursor and greener solvents. The use of another calcium source, such as calcium acetate monohydrate, was already reported in other hybrid systems.^8,16^ The different solvents and anions from calcium acetate (CH_3_COO^–1^) are expected to change the solution properties and consequently influence their behavior during electrospinning. Likewise, the distinct calcium sources can lead to varying degrees of calcium incorporation into the hybrid structure, affecting homogeneity, mechanical properties, and bioactivity. An initial optimization of electrospinning parameters, as distance from needle tip to collector, applied voltage, and flow rate, was conducted. The objective was to determine which ES parameters ensure a stable spinning of the current system and, consequently, achieve a uniform fiber diameter and beadless meshes. Afterward, two hybrid membranes with distinct fiber diameters were selected for further analysis to investigate the influence of fiber diameter on the chemical, physical, and bioactive properties of the electrospun hybrid mats. Cytocompatibility studies were also performed to compare the hybrid membranes with pure PCL.

## 2 Materials and Methods

### 2.1 Materials

Tetraethyl orthosilicate (TEOS, 99.5%, Sigma-Aldrich, Germany), trimethyl borate (TMB, 99.0%, Fluka Honeywell), and calcium acetate monohydrate (CaAc, C_4_H_8_CaO_5_, >99.0%, Sigma-Aldrich) were used to prepare the inorganic solution. The solvent and catalyst used were isopropyl alcohol (IPA, C_3_H_8_O, >99.9%, JMS, Portugal) and hydrochloric acid 25% (HCl 25%, PanReac ApplicaChem, Germany). Poly­(ε-caprolactone) (PCL, *M*_w_ 80 kDa) from Sigma-Aldrich (Germany) and glacial acetic acid (AA) from VWR Chemicals (Germany) were used to prepare the polymer solution. A phosphate-buffered saline (PBS) from Sigma-Aldrich (Germany) was used.

### 2.2 Preparation of the Solutions

Sol–gel solution was prepared by dissolving calcium acetate in isopropanol; afterward, HCl 25%, TMB, and TEOS, were added under continuous magnetic stirring at room temperature (RT) for 3 h and 20 min in an ultrasonic bath. The molar ratio between the components was TEOS/TMB/CaAc/HCl/IPA = 1:0.1:0.3:0.68:7.5. Simultaneously, a polymer solution of PCL in AA, at 15% w/v concentration, was prepared and stirred using the same conditions as the sol (inorganic solution). Subsequently, the two solutions were combined, using an organic–inorganic weight ratio of 77/23 wt %, stirred for 1 h, and electrospun.

### 2.3 Solution Analysis: Conductivity

The rheology properties of the solutions were analyzed using a Thermo Scientific Haake iQ rheometer equipped with a cylindrical cone (CC25° DIN/Ti). Rheology experiments were conducted at room temperature (25 °C) to simulate lab conditions, with shear rates ranging from 1 to 1000 s^–1^. The viscosity value was obtained at 1000 s^–1^ shear rate, and the results are shown in the Supporting Information, S.I. (Figure S1). The conductivity of the neat PCL and O/I hybrid solutions was measured using a Cond probe InLab 720, from Mettler Toledo (U.K.).

### 2.4 Fabrication of Membranes by Electrospinning

The PCL and O/I hybrid membranes were produced in an electrospinning machine (FLUIDNATEK LE-50, Bioinicia, Spain), equipped with a high-voltage power supply and a syringe pump. The prepared solutions were transferred into 5 mL plastic syringes fitted with 16-gauge metallic needles. A metallic drum covered with aluminum foil was used as the collector with a rotation speed of 200 rpm. Optimization of the electrospinning parameters was conducted to identify the optimal conditions for the hybrid solution. The parameters tested included needle tip-to-collector distances (TCD) of 12–18 cm, flow rates of 125–200 μL·h^–1^, and applied voltages of 14–20 kV. Sample designations followed the *X*–*Y*–*Z* format, where *X* denotes the tip-to-collector distance in cm, *Y* denotes the flow rate in μL·h^–1^, and *Z* indicates the voltage in kV. The optimization tests were run for 30 min to 1 h each. Electrospinnability of the solution was assessed by observing the stability of the cone-jet and by confirming the nonexistence of droplets and beads on scanning electron microscopy (SEM) images of the formed fibers.^10^ In all experiments, the temperature and air humidity ranged from 22 to 25 °C and 35 to 40%, respectively. After optimization tests, two uniform and beadless mats with two distinct fiber diameters were chosen.

### 2.5 Characterization of Electrospun Membranes

#### 2.5.1 Morphological Characterization

The material morphology was assessed using SEM (Hitachi SU70, Japan), with 15 kV of accelerating voltage and 1.0k and x6.0k magnifications. Before SEM analysis, all samples were sputter-coated with carbon. The average fiber diameter was evaluated using ImageJ software by examination of 100 random fibers for each sample. Fibers with beads were discarded from diameter calculations. An elemental map of the hybrid membranes was obtained by energy-dispersive X-ray spectroscopy (SEM-EDS).

#### 2.5.2 Chemical-Structural Analysis

Scanning transmission electron microscopy (STEM) (Hitachi HD2700, Japan) was used to examine the structure of the hybrid nanofibers and confirm the distribution and size of the inorganic components within the fibers. Fibers were directly electrospun onto copper grids for 10 s, and then, the samples were coated with a carbon film. A chemical assessment was performed by attenuated total reflectance Fourier transform infrared (ATR-FTIR) spectroscopy, on a Bruker Tensor 27 spectrometer (Massachusetts). The spectra were captured in the interval of 400–4000 cm^–1^, using a 2 cm^–1^ resolution and 250 scans. The sample’s structure was analyzed using ^1^H magical angle spinning nuclear magnetic resonance (MAS NMR), with a 4 mm probe and 15 kHz rotation speed. The chemical structures of boron were studied using solid-state ^11^B magical angle spinning nuclear magnetic resonance (MAS-HAHN-ECHO NMR), with an MAS probe of 4 mm and a sample rotation speed of 30 kHz. A Hahn echo sequence was employed for data acquisition. A Bruker Avance-700 NMR spectrometer was used in both nuclei.

#### 2.5.3 Thermal Analysis

The thermal behavior of PCL and O/I hybrids was analyzed by thermogravimetric analysis (TG) and differential scanning calorimetry (DSC). TG analysis was performed in Hitachi STA300 equipment from room temperature to 800 °C, at a heating rate of 10 °C·min^–1^ under a nitrogen atmosphere. The weight loss curves were used to determine the residual weight and thermal stability of the membranes. From DSC, the thermal properties of the samples, namely, the crystallization temperature (*T*_c_), melting temperature (*T*_m_), melting enthalpy, and crystallinity, were investigated. Measurements were conducted on a Diamond DSC, PerkinElmer, with a heat–cool–heat program from −90 to 100 °C, using heating and cooling rates of 10 °C·min^–1^ under a nitrogen atmosphere (20 mL·min^–1^). The degree of crystallinity (χ_c_) was determined from the melting enthalpy of the first run, according to eq 1 ^6,17,6,17^

1 $$X_{c}=\frac{\Delta H_{m}-\Delta H_{cc}}{f\times \Delta H_{mo}}\times 100$$

where Δ*H*_m_ is the melting enthalpy of the sample in the heating cycle, Δ*H*_cc_ is the enthalpy of cold crystallization, Δ*H*_mo_ is the theoretical melting enthalpy of 100% crystalline PCL, which equals 134.9 J·g^–1^,^17^ and *f* is the weight fraction of PCL in the membranes.

#### 2.5.4 Mechanical Characterization

The mechanical properties of the fibrous mats were evaluated using a Shimadzu EZ-LX Test tensile testing machine (Shimadzu, Kyoto, Japan) on rectangular strip samples. The thickness of the specimens was measured with a micrometer. The tests were carried on with a 10 N load cell under a speed of 1 mm·min^–1^, at room temperature. The mechanical properties determined were derived from stress–strain (σ–ε) curves. Ten specimens (*n* = 10) of 5 mm × 50 mm dimensions were tested to calculate the mean and standard deviation for each membrane.

#### 2.5.5 Wettability Analysis

The surface wettability of PCL and the O/I hybrids was investigated by static water contact angle (WCA) to assess the hydrophilic or hydrophobic character of the membranes. WCA measurements were performed at ambient temperature using the sessile drop method with a 3 μL drop of deionized water and a tensiometer (Attension Theta, Biolin Scientific, Sweden). Three measurements were taken at different spots on each membrane at 10th second, and the average value along with standard deviation was calculated.

#### 2.5.6 *In Vitro* Degradation and Bioactivity Study

*In vitro* test was performed to assess the patch degradation behavior and bioactivity in simulated physiologic conditions. The experimental procedure and results are presented in the Supporting Information (Figures S3–S6).

### 2.6 *In Vitro* Cytotoxicity Study

For the cytotoxicity studies, the selected organic and hybrid membranes, previously sterilized by UV treatment on both sides for 30 min each, were seeded with L929 mouse fibroblasts (10^4^ cells·cm^–2^, NCTC clone 929, ATCC). Seeded membranes were cultured for 1, 3, and 7 days in α-minimal essential medium containing 10% fetal bovine serum, 50 μg·mL^–1^ ascorbic acid, 100 IU·mL^–1^ penicillin, 2.5 μg·mL^–1^ streptomycin, and 2.5 μg·mL^–1^ fungizone (all reagents from Gibco), at 37 °C in a humidified atmosphere of 5%/CO_2_ in air. Cell response was evaluated for viability/proliferation (Resazurin and MTT assays), and observation was carried out by SEM.

#### 2.6.1 Resazurin Assay

Resazurin assay is a redox-based colorimetric fluorescent analysis in which the reducing dehydrogenase enzymes present in living and proliferating cells convert the oxidized, nonfluorescent dye form (blue) into a reduced, fluorescent form, resorufin (pink). In the assay, colonized membranes were incubated with 10% v/v Resazurin solution (Sigma-Aldrich, St. Louis, MO) prepared in complete medium (described above) for 3 h and 37 °C. Cell viability was assessed on days 1, 3, and 7 during cell culture. Fluorescence intensity (excitation maximum at 530 nm and emission maximum at 590 nm) was measured in a microplate reader (Synergy HT, Biotek) with Gen5 Data Analysis. Results were expressed as relative fluorescence units (RFUs).

#### 2.6.2 MTT Assay

MTT method is based on the reduction of MTT [3-(4,5-dimethylthiazol-2-yl)-2,5-diphenyltetrazolium bromide] by the mitochondrial succinic dehydrogenase of viable proliferating cells to violet-blue formazan crystals. At each time point, the colonized membranes were incubated with MTT (5 mg·mL^–1^, Sigma-Aldrich, St. Louis, MO), for 3 h and then, the violet-blue stained cultures were imaged under a stereo microscope (Stemi 305, Zeiss, Oberkochen, Germany).

#### 2.6.3 SEM Analysis of Cell Morphology

The attachment and morphology of the fibroblasts growing on the membranes’ surface were analyzed by SEM. Samples were fixed in 1.5% glutaraldehyde solution (30 min, TAAB, Berk, England) and dehydrated in graded ethanol concentrations (50, 70, 90, and 100% ethanol) followed by critical point drying (CPD 7501, Polaron Range). Membranes, sputter-coated with gold–palladium, were observed by SEM (FEI Quanta 400 FEG ESEM/EDAX Genesis X4M; FEI Company, Hillsboro, OR).

#### 2.6.4 Statistical Analysis

Results were presented as mean ± standard deviation of three independent experiments, with three replicas each. Statistical analysis was performed by one-way analysis of variance, in combination with Tukey’s post hoc test. Values of *p* < 0.05 were considered significant.

## 3 Results and Discussion

### 3.1 Solution Analysis

Solvent selection is crucial for efficient electrospinning, especially when aiming for nontoxic and sustainable fabrication methods. PCL, known for its solubility in a variety of solvents, has shown promising results with green solvents such as acetic and formic acid (FA). This study investigated the application of single and binary solvent systems, specifically FA and AA in volume ratios of 2:1 and 1:1 (FA/AA), as well as AA alone. When FA was present in the mixture, the polymer solutions became cloudy upon the addition of the sol. Conversely, solutions with AA alone demonstrated good miscibility among the sol and the polymer, resulting in a homogeneous and transparent solution. Hence, no phase separation was detectable at the scale of the visible light wavelength. This behavior can be attributed to the acid strength (*K*_a_ values) of FA and AA, where FA (*K*_a_ = 1.77 × 10^–4^) is stronger than AA (*K*_a_ = 1.76 × 10^–5^), making it more reactive and prone to inducing phase separation.^18^ Based on these results, AA was selected, offering a 24 h handling window before gelation begins and can be electrospun. Solution viscosity is well known as a key parameter in determining the fiber morphology and size. The optimal spinning viscosities typically range from 0.1 to 21.5 Pa·s.^1^ Thus, the solution’s viscosity and viscosity change over time were determined, at 1000 s^–1^ shear rate, and the graphs are represented in S.I., Figure S1a,b, respectively. Observing the graphs in Figure S1­(a), the viscosity was approximately 1663 mPa·s for PCL and 99.3 mPa·s for the hybrid solution. This reduction in viscosity, owing to the incorporation of inorganic components, is consistent with the reports that adding silica sol decreases PCL solution viscosity, unlike bioglass nanoparticles which increase it.^11,19^ Furthermore, a solution with PCL and acid (HCl 25%), named PCL–HCl, was prepared and compared with pure PCL solution to assess if the presence of acid contributed to the observed decrease in viscosity. As shown in Figure S1, the viscosity of PCL solution remained stable over time. However, when the acid was introduced, the viscosity values decreased, as occurred in the hybrid solution. Additionally, it was not possible to electrospun the PCL–HCl solution from the start; only droplets were formed instead of fibers. Thus, this could indicate that HCl contributed to the scission of PCL molecular chains, leading to a reduction in polymer molar mass and, consequently, lowering the solution viscosity.^20^ However, when the inorganic components were added, the final solution became spinnable. Therefore, the presence of the inorganic content helped to maintain a sufficient viscosity for electrospinning. It was previously found that the ideal viscosity range for electrospinning a gelled solution was 25–35 mPa·s.^11^ According to Bhardwaj et al., the polymer type, the solvent, and the existence of salts can influence the solution conductivity (σ).^1^ PCL alone exhibited a low conductivity of σ = 0.110 ± 0.001 μS·cm^–1^, while the hybrid solution reached a conductivity of 146.88 ± 1.03 μS·cm^–1^. This goes in accordance with other reports that showed an enhancement of conductivity and, consequently, in spinning performance with the addition of ions or salts.^1,10,21^ AA and PCL are both known for their low conductivity: AA has a σ of 0.057 μS·cm^–1^ and dielectric constant, ε, of 6.20 at 20 °C, which may result in unstable spinning.^10,12^ Studies with calcium and borosilicate glass compositions have demonstrated rapid dissolution rates. Thus, the electrical conductivity of polymer solutions can increase, since Ca^2+^ and B^3+^ ions can act as charge carriers.^21,22^ Furthermore, hydrolysis-induced water formation in the system contributed to the increase of conductivity, facilitating the electrospinning process.^12^

### 3.2 Characterization of Electrospun Membranes: Effect of Electrospinning Parameters on Jet Behavior and Structure-Microstructure of Electrospun Membranes

As previously noted, electrospinning relies on the solution, process, and environmental conditions. In here, the ambient conditions were monitored. The process parameters examined included the needle tip-to-collector distance (TCD) (12, 15, and 18 cm), flow rate (125, 150, and 200 μL·h^–1^), and applied voltage (14, 17, and 20 kV). These variables were analyzed for their impact on jet stability and the uniformity of the produced membrane, specifically bead-free fibers and the absence of droplets or clusters on the membrane. The morphology was investigated using SEM imaging, and the optimal hybrid membranes were selected for further characterization.

#### 3.2.1 Jet Stability

Experiments on jet stability were conducted by adjusting the voltage and flow rate across three different TCD: 12, 15, and 18 cm. The results are shown in Figure 1 for each distance: (a) 12 cm, (b) 15 cm, and (c) 18 cm and were classified into three behaviors: stable, unstable, and dripping. Stability was defined by the formation of a single jet and Taylor cone. At TCD of 12 cm, Figure 1(a), dripping occurred at higher flow rates combined with lower voltages, which suggests that an excess of solution at the needle tip could not be stretched efficiently by the electric field. Increasing the voltage reduced droplet formation, but the jet becomes unstable, leading to multiple jet formation. Nevertheless, low flow rates also failed to maintain a stable jet as the voltage increased. According to the literature, highly conductive solutions are unstable under strong electric fields, leading to jet bending.^1^ At TCD of 15 cm, Figure 1(b), this setup showed inconsistent jet behavior, showing both dripping and instability of the Taylor cone. Despite these variations, fibers were successfully produced. Extending the distance to 18 cm improved the jet’s stability at lower flow rates, Figure 1(c). Dripping behavior was noted when the flow rate exceeded 125 μL·h^–1^; however, increasing the voltage contributed to better jet stability (*e.g.*, 200 μL·h^–1^ and 20 kV).

**Figure 1:**
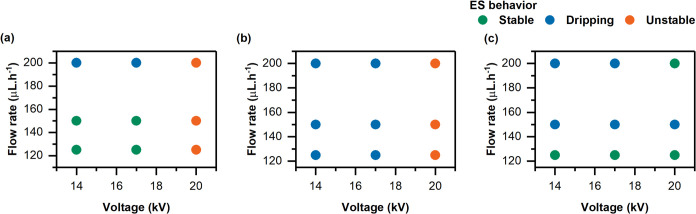
Electrospinning behavior for TCD of (a) 12 cm, (b) 15 cm, and (c) 18 cm at different flow rates (125, 150, and 200 μL·h^–1^) and voltages (14, 17, and 20 kV).

#### 3.2.2 Morphology Analysis

SEM micrographs were used to assess the homogeneity of the resulting membranes, the absence of defects or clusters, beads, and droplets on the electrospun meshes, and the uniform fiber diameter distribution. Figure 2 presents the SEM images of the hybrid membranes electrospun at 12 cm TCD, for the three different flow rates (125, 150, and 200 μL·h^–1^) and voltages (14, 17, and 20 kV). As seen from the images, uniform and continuous fibers with no inorganic particle clusters or defects were formed under all tested parameters. In addition, bead-free fibers were mainly obtained. For this TCD, a broad range of flow rates and voltages was possible to give a defect-free membrane, *i.e.*, uniform fiber diameters, bead-free fibrous morphology, and the absence of defects. Details for other TCDs can be found in the Supporting Information (Figure S2).

**Figure 2:**
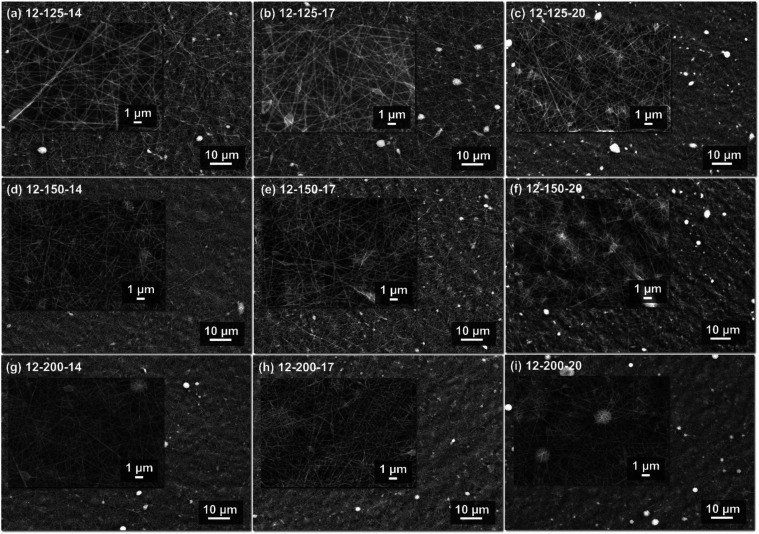
SEM micrographs, at ×1.0k and ×6.0k magnification, of membranes electrospun at 12 cm TCD with flow rates of 125 (a–c), 150 (d–f), and 200 (g–i) μL·h^–1^, and voltages of 14 (a, d, g), 17 (b, e, h), and 20 kV (c, f, i).

Overall, an increase in flow rate typically required higher voltages, but the higher flow rates often resulted in beaded fibers.^12^ This phenomenon has been attributed to a reduction in the droplet’s surface net charge as the flow rate increased, resulting in weaker stretching forces at constant voltage.^12^ Therefore, higher voltages are necessary at elevated flow rates to generate sufficient stretching forces for smooth fiber formation. However, according to the literature, higher flow rates and voltages may lead to bead formation owing to the shorter time available for the solvent to evaporate before being collected.^1^ In the case of the influence of TCD, studies indicate that TCDs that are too short (*e.g.*, 10 cm) or too long (*e.g.*, 20 cm) have a greater tendency to form beads.^1^ Though, in this study, the hybrid solution produced continuous, bead-free fibers both at 12 and 18 cm distances, by adequately changing the flow rate and voltage, as observed in Figures 2 and S2. In general, hybrid nanofibrous membranes displayed uniform morphology, beadless fibers, and no visible inorganic particles. In some SEM images, *e.g.*, Figure 2(i), bright areas appeared, which can be attributed to charged regions with a high concentration of elements. This outcome has been previously reported by Allo et al. when adding TEOS and CaCl_2_·2H_2_O to PCL solutions.^11^

Fiber diameter analysis showed that increasing TCD allowed for thinner fibers since fibers could adequately dry before reaching the collector. However, at an 18 cm TCD, larger fiber diameters were also formed, probably due to the decrease in the electrical force acting on the solution. This happened for the case of 125 μL·h^–1^ flow rate with voltages of 14 and 17 kV. According to published research, a minimum distance is necessary to ensure that the fibers have enough time to dry before being collected. Some reports even showed that using small distances have yielded fibers with low average diameters.^1^ This was corroborated by the present study, where fiber diameters as low as 62.7 nm were achieved at a 12 cm TCD. In terms of voltage influence, for the most part, when the voltage was increased, the fiber diameter decreased owing to the increase of surface charge density and stretching force on the jet.^1^ This trend was clear at low flow rates (125 μL·h^–1^), where thinner fibers were produced for higher voltages. However, when the jet was unstable or dripping occurred, this tendency was not detected, for instance, at flow rates of 150 and 200 μL·h^–1^ with 20 kV of voltage. Studies have suggested that higher voltages can also promote multiple jet formation at the needle tip, leading to larger fiber diameters and a greater probability of bead formation.^1,23^ Thus, a balance between flow rate and voltage is necessary for stable spinning; an increase of flow rate imposes a corresponding increase in voltage. For this hybrid system, better spinning and mesh morphology are obtained at 12 cm TCD, low flow rates (125–150 μL·h^–1^), and voltages (14–17 kV). Based on these findings, two electrospinning conditions with different fiber diameters were chosen for extended spinning and further analysis: 12–125–17 and 12–150–14 conditions.

##### 3.2.2.1 PCL *vs* Hybrid Membranes

The influence of adding inorganic components to the polymeric solution was evaluated by comparing the morphology and fiber diameter of neat PCL and hybrid electrospun meshes (Figure 3). Figure 3(a), at ×6.00k magnification, shows that the neat PCL sample consisted of fibers with a broad diameter distribution and some large beads. The mats are composed of randomly dispersed fibers with an average fiber diameter of 684.46 ± 391.57 nm. In contrast, the hybrid electrospun meshes exhibited greater uniformity, reduced bead formation, and a narrower fiber diameter distribution, as shown in Figure 3(b,c) at ×6.00 and ×15.00k magnification. For this hybrid system, the average fiber diameter was in the nanometer range, 65.29 ± 8.77 and 116.32 ± 20.57 nm for 12–125–17 and 12–150–14 samples, respectively. Homogeneous mats can be attributed to the inclusion of an inorganic sol. Literature supports the idea that PCL solutions with only formic acid or acetic acid can be electrospun in a stable state, with instability occurring due to distinct dielectric constants.^24^ A mixture of both solvents is often recommended to achieve intermediate polarity and maintain a steady spinning condition.^10^ Nevertheless, some authors have reported that PCL solutions, dissolved in acetic acid and containing BG nanoparticles, were successfully electrospun,^5,25^ despite occasional particle agglomeration.^14,17^ In the current study, a homogeneous and clear hybrid solution was prepared *in situ*, using AA as the green solvent of the organic part, and was able to be electrospun. This was possible due to the presence of conductive additives, such as metallic ions, which can enhance the conductivity of weak polar solvents, thereby improving spinnability and yielding a more uniform membrane morphology. Likewise, the rise in electrical conductivity of the solution has been shown to notably reduce the diameter of electrospun fibers.^1,2,10,24^ Zong et al. reported that small fiber diameters, varying from 200 to 1000 nm, were obtained when incorporating ionic salts, such as NaCl.^25,26^ This phenomenon was also seen in current research, where the hybrid solution produced membranes with thinner fibers compared with the neat PCL solution. Therefore, these findings emphasize that, besides the ES parameters, solution conductivity and viscosity can affect the fiber diameter through the introduction of fillers.^8^

**Figure 3:**
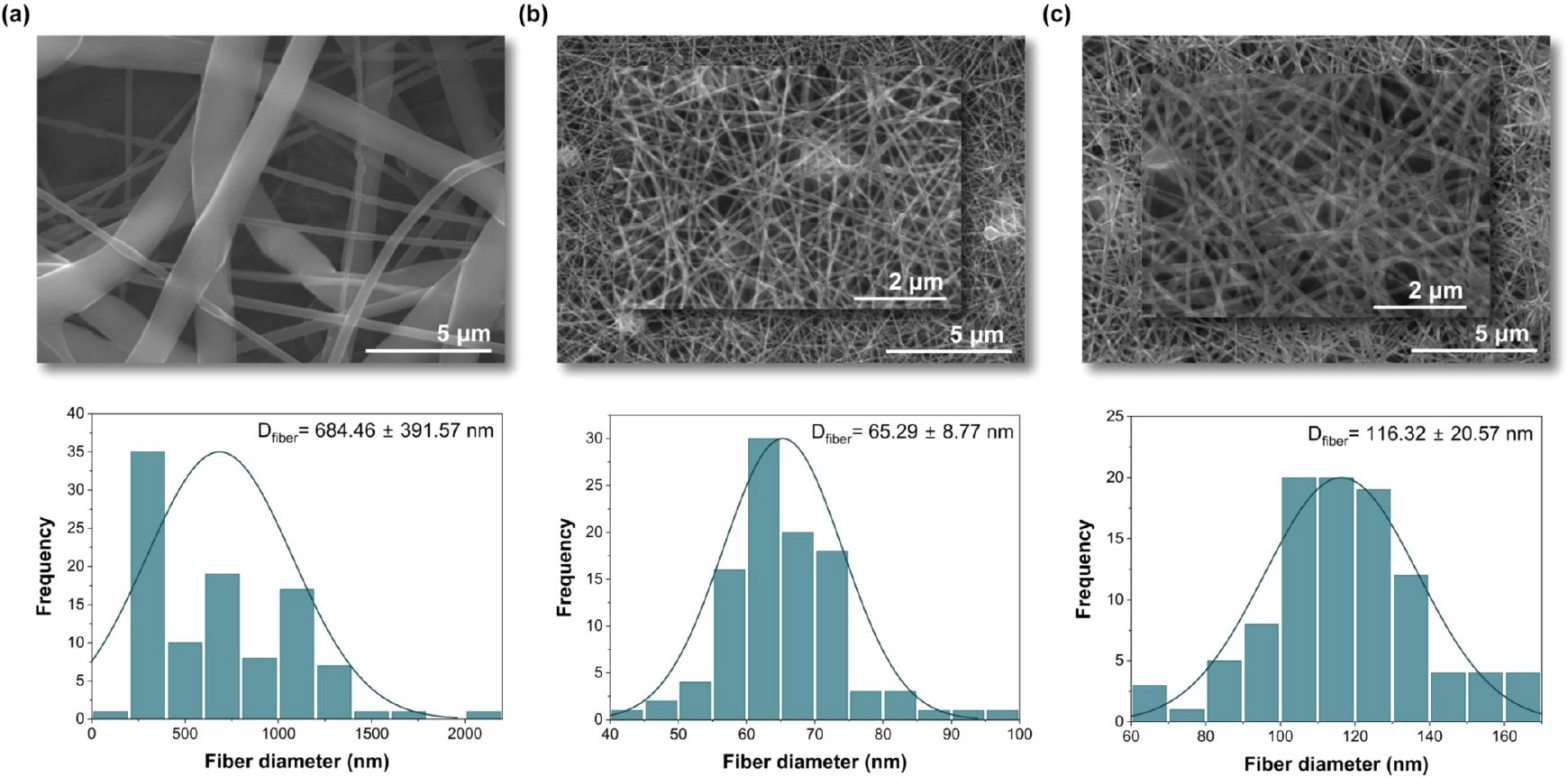
SEM micrographs of (a) PCL, (b) 12–125–17, and (c) 12–150–14 hybrid membranes and respective fiber diameter distribution with the mean and standard deviation of the samples identified.

#### 3.2.3 Chemical-Structural Analysis

Considering the element distribution in the fibers, EDS elemental mapping was performed on the hybrid membrane 12–125–17 using both SEM (Figure 4a) and STEM (Figure 4b) analyses. As observed in both cases, silicon (Si) and calcium (Ca) ions were homogeneously distributed throughout the nanofibers. This uniform distribution confirms the successful integration of the inorganic components into the polymeric matrix. In STEM analysis, Figure 4(b), the presence of Si (green micrograph) and Ca (red micrograph) ions at the nanometer scale was confirmed, providing additional evidence of their consistent incorporation.

**Figure 4:**
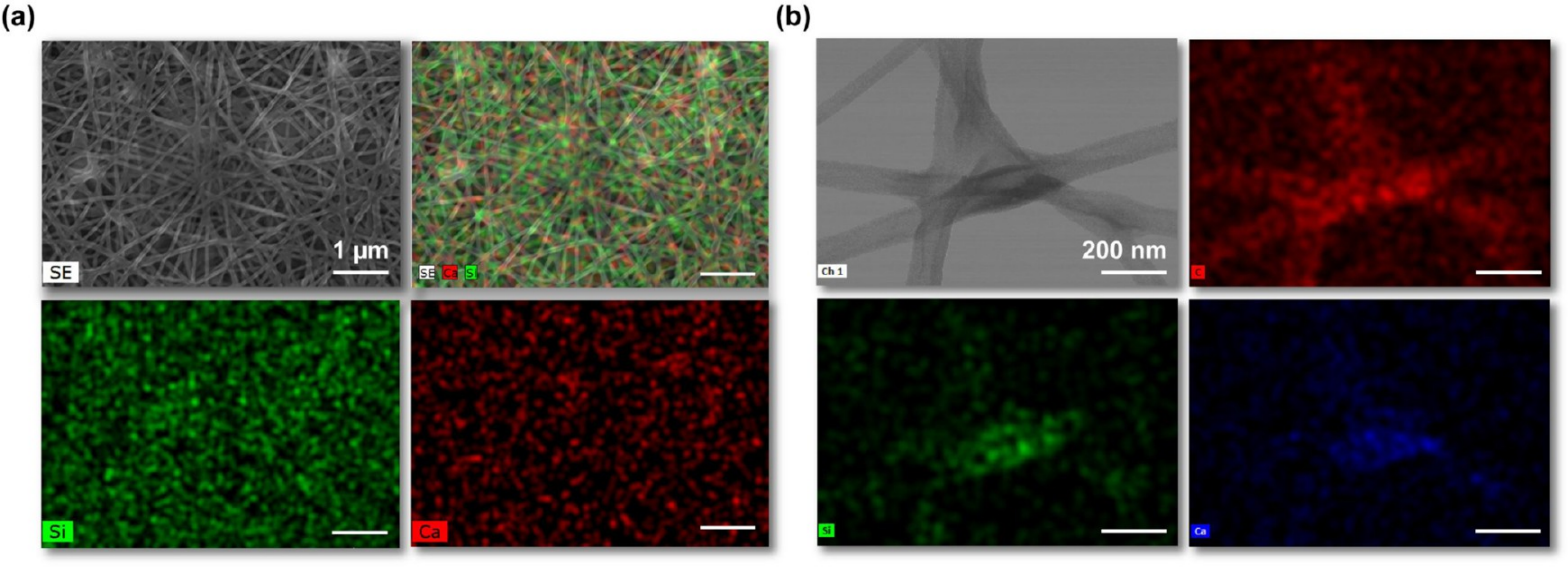
(a) SEM micrograph and EDS elemental mapping of a combination of Si (green) and Ca (red) atoms, Si and Ca atom distribution, scale bar 1 μm; (b) STEM micrograph and EDS elemental mapping of C (red), Si (green), and Ca (blue) atoms, scale bar 200 nm.

##### 3.2.3.1 ATR-FTIR

ATR-FTIR analysis was performed to identify the chemical interactions between the two phases and analyze the influence of the ES parameters on the chemical structure. Figure 5 compiles the IR spectra of PCL and hybrid 12–125–17 and 12–150–14 compositions in the wavenumber ranges of (a) 4000–400 cm^–1^ and (b) 1600–400 cm^–1^. The peak positions of PCL and hybrid membranes are listed in Table 1 along with the respective band assignments. The spectra of both neat PCL fibers and hybrid fibers are primarily characterized by the main PCL bands, such as CH_2_ asymmetric (ν_a_) and symmetric (ν_s_) stretching vibrations at approximately 2945 and 2867 cm^–1^, respectively^11,27,28^ (Figure 5a). A characteristic peak of PCL appears at 1722 cm^–1^ and is attributed to the carbonyl (C=O) stretching band of the ester group.^29,30^ For the hybrids, this peak tends to broaden and shift for higher wavenumbers (*ca.*1724 cm^–1^). This change can be attributed to changes in the structure due to the incorporation of inorganic parts. Since PCL is a semicrystalline polymer, the crystalline and amorphous regions can be identified at 1294 and 1160 cm^–1^, being assigned to C–O and C–C stretching vibrations.^6,28^ In the hybrids, the latter band tends to shift to higher wavenumbers, as identified in the spectra of Figure 5(b).^5,31^

**Figure 5:**
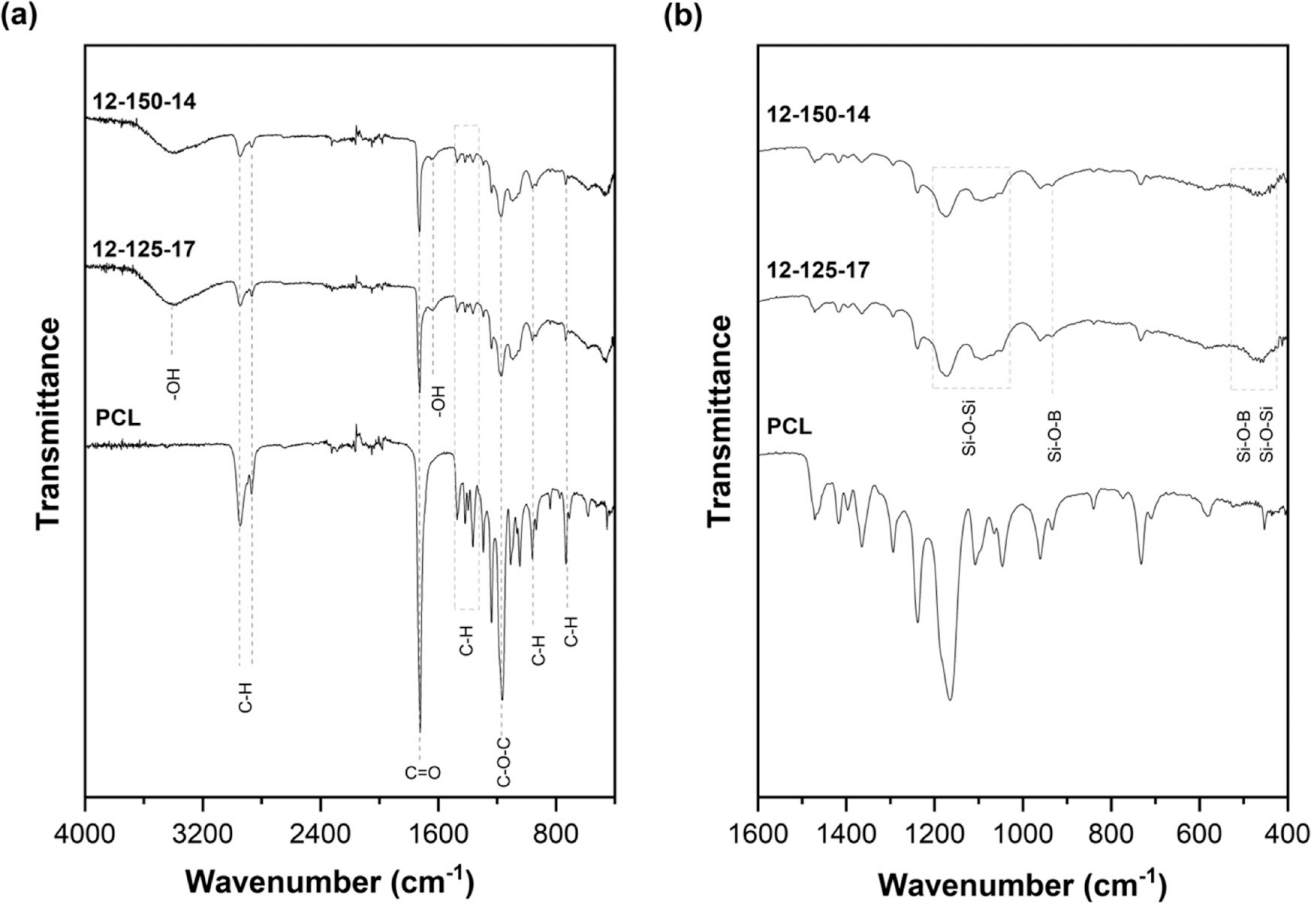
ATR-FTIR spectra of electrospun membranes in the (a) 4000–400 cm^–1^ region and (b) 1600–400 cm^–1^ region and respective band identification.

**Table 1:** FTIR Band Assignment and Peak Position for PCL and Hybrid Membranes

band assignment	PCL	12–150–14	12–125–17	refs
–OH stretching		3416	3405	^11,29,31^
–CH_2_ asymmetric stretching	2950	2946	2947	^6,11,27^
–CH_2_ symmetric stretching	2867	2867	2869	^6,11,27^
C=O stretching	1722	1723	1724	^6,11^
–OH bending		1637	1640	^8,11,30^
–CH_2_ bending	1471	1471	1471	^6,27,35^
–CH_3_ bending	1419	1419	1418	^5,27^
–CH_2_ bending	1398	1398	1398	^6^
–CH_2_ bending	1366	1364	1366	^6^
C–O, C–C stretching	1294	1294	1294	^6,20^
C–O–C stretching	1239	1239	1239	^6,21^
C–O–C, C–C stretching, Si–O–Si stretching	1160	1168	1171	^6,21,28^
C–O–C symmetric stretching	1108	1105	1107	^6,28^
Si–O–Si asymmetric stretching		1087	1087	^28,32,37,38^
C–O–C stretching/–CH_2_ bending, Si–O–Si	1047	1048	1048	^6,28,8^
Si–OH asymmetrical stretching	962	961	961	^28,32,37,39^
Si–O–B stretching		935	935	^34^
Si–O–Si bending		794	800	^19,28,33^
–CH_2_ stretching, B–O–B bending	732	731	733	^8,11^
Si–O–B bending		669	668	^8,18,40,41^
Si–O–B stretching		468	470	^8^
Si–O–Si bending		453	454	^8,28,35^

Additionally, the spectra of the hybrids show typical bands of adsorbed water molecules, approximately at 1640 cm^–1^, which are ascribed to the bending vibration of −OH groups.^8,11,31,32^ One feature of sol–gel glasses is high porosity which enables them to host water molecules, and the presence of silanol groups in the glass network is responsible for this property.^32^ The peak at 3400 cm^–1^ is ascribed “to the hydroxyl stretching vibrations of the self-associated silanol groups, and the width of the peak reflects the wide frequency distribution of the hydrogen-bonded −OH groups.” ^11,30^ According to Allo et al., the “broadening of this peak indicates the formation of hydrogen bond interactions between Si–OH and carbonyls” of the polymer.^11^ Likewise, the slight shoulder that develops at 1700 cm^–1^ in the hybrids can be representative of this hydrogen bond.^11^ The distinctive bands of boron and silica bonds are not clearly visible in the hybrid’s spectra, owing to the overlap of the PCL band. Though, observing Figure 5(b), differences are detected in the configuration, particularly in the 1200–1000 cm^–1^ region, which are ascribed to asymmetric stretching of Si–O–Si bonds in the silica group.^31^ This region is composed of peaks at 1090 and 1072 cm^–1^ attributed to Si–O–Si stretching mode.^11^ Other typical bands of Si–O–Si vibration modes are at 960, 795, 570, and 453 cm^–1^.^19,27,31,32^ However, here several bands are overlapped by PCL bands. The band at 795 cm^–1^, assigned to the bending Si–O–Si vibration, is characteristic of ring structures in the glass matrix.^19,31,32^ In addition, borosiloxane (Si–O–B) bonds and boron bonds, B–O–B, typically form when boron is added to the silica network. The standard peaks of B–O–B linkages are around *ca.* 1149 and 731 cm^–1^.^8^ Some authors have reported the existence of Si–O–B bonds at 940–915, *∼*670, and ∼470 cm^–1^, being attributed to stretching and bending vibrations.^18,33,34^ Although not evident, due to the intersection of PCL bands, a change in the configuration was observed.^42,43,45,47^

##### 3.2.3.2 NMR Analysis

PCL and hybrid 12–125–17 and 12–150–14 samples were studied by ^1^H MAS NMR and the results are presented in Figure 6(a). The peaks observed at 1.4, 1.6, 2.3, and 4.0 ppm can all be assigned to hydrogens in the backbone structure of PCL (see the (c), (d, b), (e), and (a)) positions in Figure 6(a).^48^ Regarding the spectra obtained for hybrid materials, it appears that all of these peaks are broader and present a downfield, which may be related to a loss of mobility of the methylene groups, caused by the proximity of the inorganic structures.^44^ Despite the broadness of the peaks, it is possible to observe two small shoulders in the spectra of sample 12–150–14 (dashed circles) possibly assigned to silanol (*ca.* 2.1 ppm)^44^ and boroxol groups (*ca.* 3.8 ppm).^49^ According to published reports, the presence of calcium in the composition explains the appearance of a narrow peak at 4.8 ppm, which is being ascribed to physiosorbed water molecules.^8,50,51^

**Figure 6:**
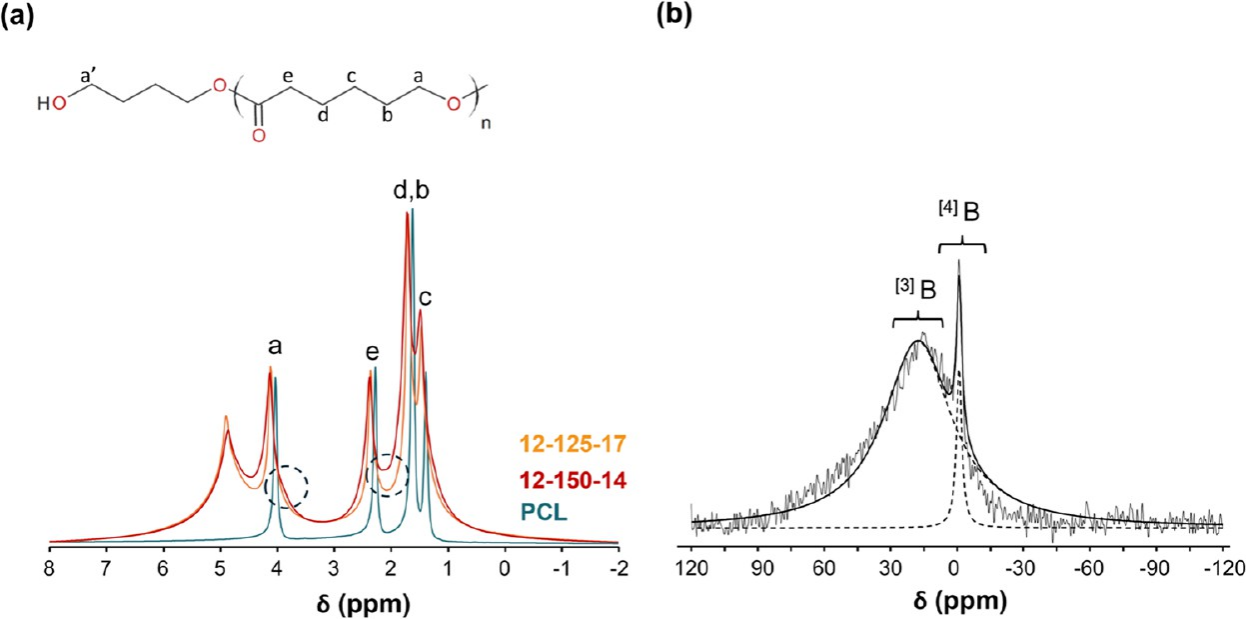
(a) ^1^H NMR spectra of PCL and hybrid membranes. Dashed circles indicate the presence of silanol (*ca.* 2.1 ppm) and boroxol (*ca.* 3.8 ppm) groups; (b) ^11^B NMR HAHN-ECHO spectrum of the hybrid 12–125–17 sample.

^11^B MAS NMR technique was used to study hybrid compositions. This technique has been widely used to study borosilicate glasses and boron-containing hybrids. Figure 6(b) illustrates the spectrum of the 12–125–17 hybrid sample. In the spectrum, two peaks appearing between −1 and 18 ppm are correlated with distinct groups of B structural units. Hence, this reveals the presence of different boron structural arrangements. The first peak near −0.66 ppm can be assigned to tetragonal BO_4_ (^[4]^B) groups, corresponding to B–O–Si bonds in a tetrahedral configuration. This configuration has been previously noted in borosilicate glasses and hybrids, being correlated to the existence of calcium in the system, which functions as a modifier and whose field strength influences boron coordination.^8,9,52–54^ The second broad peak at *ca.* 15.54 ppm can be ascribed to trigonal BO_3_ (^[3]^B) units connected to SiO_4_ tetrahedra. Although it is challenging to accurately identify the various components, due to the broad peak and significant signal overlap, they can still be ascribed to trigonal B sites.^46^ This signal is near the one documented in the literature for ^11^B resonance, δ = 13 ppm, and serves as the fingerprint of borosiloxane bond formation.^46,54–56^

In sol–gel chemistry, Si–O–B bonds are known to lack stability during the gelation step due to the hydrolytic attack.^8^ The condensation reactions involving B–OH and Si–OH groups can produce water, which may hydrolyze back the borosiloxane bonds.^8^ Works from Irwin et al. demonstrated that in gels dried at ambient conditions, the majority of boron exists as boric acid that is hydrogen-bonded to the silica, and a small portion forms Si–O–B groups.^57,58^ However, other authors have reported that the amount of borosiloxane bridges increased when the hybrid siloxane network contained organic groups, as methyl groups.^46^ This effect was attributed to the hydrophobic nature of these groups, which, in proximity to the borosiloxane bridges, “could serve to act as an *in situ* protection of the Si–O–B bonds from the water attack”.^59^ The same was confirmed by other authors in PDMS-SiO_2_–B_2_O_3_ systems.^8,34,37^ Hence, literature proposes that the stability of Si–O–B bonds could be ascribed to either (1) increased resistance of these bonds to hydrolysis or (2) the formation of borosiloxane bridges that remain incompletely hydrolyzed in the final steps of the process.^46^ In the present study, the second hypothesis can be the reason why the fast process applied did not allow enough time for the water formed during condensation to hydrolyze back the borosiloxane bonds, resulting in stable Si–O–B groups in the final material. Although Si–O–B bonds were not clearly identified in the IR spectra, the NMR results showed the presence of these bonds. Thus, even in low-pH solutions, the crystallization of B­(OH)_3_ was not encouraged and boron atoms participated in the creation of borosiloxane bridges.^46^

#### 3.2.4 Thermal Analysis

The thermogravimetric (TG) and derivative thermogravimetry (DTG) of PCL pellets, neat PCL, and O/I hybrid membranes are present in Figure 7a,b, respectively. Table 2 summarizes the data obtained from the curves. TG curves demonstrate weight loss during the heating process, where thermal stability and percentage of inorganic residues are revealed. Observing the TG curves, Figure 7(a), of PCL pellets and electrospun PCL membrane, a similar thermal behavior is noticed, occurring a mass loss around 300–500 °C. In this interval, the polymer was degraded into “CO_2_, hexenoic acid, and ε-caprolactone molecules.”^6^ From the derivate of weight loss, Figure 7(b), the raw PCL pellets reaches a maximum rate degradation at *ca*. 410.5 °C, being total degraded *ca*. 490.0 °C, while the PCL mesh exhibited two steps of degradation, involving the “decomposition of low-crystallinity PCL and high-crystallinity PCL”.^60^ The corresponding DTG curves present inflection temperatures at 340.5 and 411.5 °C.^5^ Regarding the hybrids, two stages of thermal decomposition were observed in the TG curves, Figure 7(a). The first weight loss occurred below 200 °C and, as already observed by other researchers, it is related to the elimination of water, remaining solvents as well as the breakdown of unreacted and thermally unstable constituents.^9,11,61^ The degradation of PCL is responsible for the second weight loss that was observed in the interval of *ca.* 200–500 °C. From the DTG curves (Figure 7b), the hybrid samples 12–125–17 and 12–150–14 present their inflection temperatures of stage II at 352.3 and 348.5 °C, respectively. Thus, a decrease in the decomposition temperature occurs when inorganic components are incorporated into the polymeric matrix. The same was observed by Ding et al. in organic–inorganic hybrid compositions containing silica and calcium, where calcium brings forward the weight loss.^28^ Besides, PCL and hybrid electrospun membranes present different fiber diameters, which could be difficult to compare as well, since fiber diameters can affect thermal decomposition. Likewise, the final residual weight at 800 °C is presented in Table 2, corresponding to 0, 2.3, 19.0, and 16.2 wt % for electrospun raw PCL, electrospun PCL, 12–125–17 and 12–150–14 hybrid membranes, respectively. In hybrid samples, the inorganic residue values are close to the theoretical ones.

**Figure 7:**
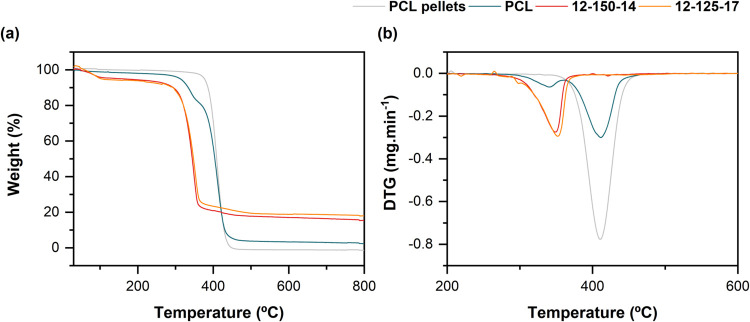
(a) TG and (b) DTG curves of raw PCL, electrospun PCL, and O/I hybrid membranes.

**Table 2:** Thermal Properties of PCL Pellets, Electrospun PCL, and Hybrid Samples Assessed by TG and DSC

	Stage I	Stage II						
composition	weight loss (%)	inflection *T* (°C)	weight loss (%)	residual mass (%)	*T*_c_ (°C)	*T*_m_ (°C)	Δ*H*_m_ (J·g^–1^)	χ_c_ (%)
PCL pellets		410.5	100.0		22.4	65.8	76.4	56.6
PCL		411.5	97.7	2.3	27.3	61.2	68.3	50.6
12–150–14	5.3	348.5	79.4	16.2	30.7	61.9	57.5	55.7
12–125–17	5.9	352.3	76.2	19.0	31.9	60.8	46.7	45.2

Figure 8 presents the DSC profiles of the first heating (Figure 8a) and cooling (Figure 8b) cycle of PCL pellets, electropun PCL, and O/I hybrid membranes. The first heating cycle curves were considered since they depict the “melting behavior of the sample that crystallized from the solution and electrospinning process.”^28^ The thermal properties of each sample are shown in Table 2. The PCL pellets present a higher melting temperature, enthalpy, and crystallinity than the electrospun PCL, but a lower crystallization temperature. According to Cipitria et al., when the polymer is submitted to the electrospinning process, a quick solvent evaporation may occur, not allowing enough time for crystal nucleation and therefore leads to a deficient crystal structure.^62^ This way, the crystallinity tends to decrease when the material is electrospun. From the cooling scan (Figure 8b), it was possible to observe that the crystallization temperature (*T*_c_) increased with the inclusion of inorganic components, being close to the reported ones.^6,63^ Other studies have reported the same when silica and calcium were incorporated into the organic matrix.^28^ However, for the melting temperature (*T*_m_), no significant differences were observed, while a change in the melting enthalpy was detected, which shifted to lower values. This reduction of ΔH_m_ is rational given the smaller polymer content in the hybrid compositions when contrasted with the pure PCL membrane.^6,64^ Likewise, the calculated crystallinity degree of the materials has revealed a change when inorganic components were added. An increase was seen in the 12–150–14 sample, while a decrease was seen in the 12–125–17 sample. In the literature, different data have been reported and many factors, for instance, processing technology, inorganic content, and the interactions between parts, can affect polymer crystallization. Some authors have reported that ceramic fillers, *e.g.*, nanoparticles, could act as a nucleation agent and promote heterogeneous nucleation and crystallization process.^6,28,63^ Nevertheless, other studies have reported a decrease in the polymer’s crystallinity, particularly for considerable amounts of silica.^28^ Ding and co-workers explained this phenomenon due to the hydrogen bonds connecting the two phases, which may limit crystal formation and inhibit the polymer molecules’ movement. Among the hybrids, the sample with a higher fiber diameter, 12–150–14, presented higher crystallinity than the one with a lower, 12–125–17. Thus, it can be assumed that the hybrid samples have different degrees of cross-linking. This difference is expected to affect their mechanical properties.

**Figure 8:**
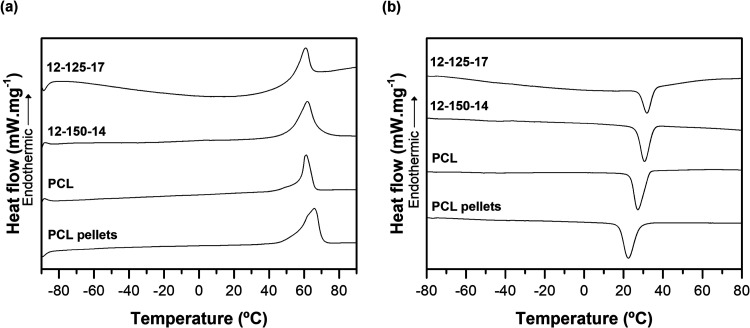
DSC thermographs of raw PCL, PCL, and hybrid electrospun membranes on the (a) heating and (b) cooling cycles.

#### 3.2.5 Mechanical Properties

Tensile tests were conducted on the electrospun fibrous membranes. Figure 9 illustrates a representative stress (σ)–strain (ε) curve for each composition, and Table 3 displays the mechanical properties of the samples. As observed in Figure 9, the neat PCL membrane exhibited a high degree of deformation, typical of ductile materials, whereas the hybrid membranes revealed similar behaviors with a transition from elastic to plastic during fracturing. From Table 3, it is possible to see that the introduction of an inorganic component in the polymer solution led to an increase of Young’s modulus (*E*) and tensile strength, particularly for the hybrid with a lower fiber diameter, and a decrease of the strain at break (ε). The PCL membrane presented an elastic modulus (*E*) of 24.1 ± 10.7 MPa and a tensile strength of 6.5 ± 0.6 MPa. In contrast, the O/I hybrids 12–150–14 displayed values of 117.0 ± 17.4 MPa for *E* and 4.6 ± 1.2 MPa for tensile strength; while the 12–125–17 sample exhibited higher values of *E*, 235.5 ± 35.4 MPa, and 10.9 ± 2.5 MPa for tensile strength. These findings are consistent with the existing literature, which states that adding an inorganic component to the organic matrix will result in an enhancement of the material’s strength and reduce its elongation.^65^ Likewise, it was observed that electrospinning conditions controlled the fiber diameter and consequently affected the mechanical properties. The 12–125–17 sample with a fiber diameter of 65.29 ± 8.77 nm presents a higher tensile modulus and strength than the 12–150–14 sample with a fiber diameter of 116.32 ± 20.57 nm. The Young’s modulus and tensile strength increased with decreasing fiber diameter. Therefore, the enhancement of the mechanical properties of the hybrid meshes can be ascribed not only to the integration of silica, boron, and calcium phases within the PCL composition but also to the nanoscale structure. It is believed that the organized nanostructure of the hybrid helps to reinforce the fibrous membrane.^61^ Authors have inferred that the ultimate tensile strength of nanofibers was directly related to the reduction in the fiber diameter, which contributes to an increase in fiber packing density.^66,67^ Besides, the absence of defects in the hybrids can possibly improve the material’s mechanical performance, since homogeneous and individualized fibers were obtained. Although the present results agreed with the literature when silica xerogel is included, the values obtained in the present study for Young’s modulus were significantly higher.^19,61,63^

**Figure 9:**
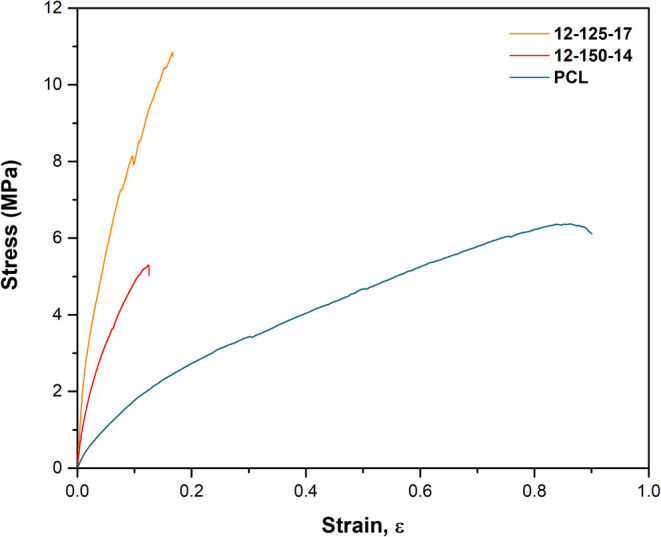
Representative stress–strain curves for PCL, 12–150–14, and 12–125–17 hybrid samples.

**Table 3:** Tensile Properties of Electrospun Membranes: PCL, 12-150-14, and 12-125-17 Hybrids^a^

composition	*E* (MPa)	ultimate tensile strength (MPa)	strain at break, ε
PCL	24.1 ± 10.7	6.5 ± 0.6	0.85 ± 0.044
12-150-14	117.0 ± 17.4	4.6 ± 1.2	0.14 ± 0.044
12-125-17	235.5 ± 35.4	10.9 ± 2.5	0.15 ± 0.048

a Data are reported as average ± standard deviation.

#### 3.2.6 Surface Wettability

The water contact angle measurements of each composition are represented in Figure 10. As is known, the surface hydrophilic nature of materials increases as WCA decreases. PCL exhibited a hydrophobic behavior, with a WCA of 124.4 ± 1.4°, which is consistent with previous reports.^36,67^ In contrast, the hybrid fibrous membranes showed reduced water contact angles of 37.0 and 45.0° for 12–150–14 and 12–125–17 samples, respectively. The hydroxyl groups in the inorganic phase may improve the wettability of the fiber mesh.^36^ As already reported, the hydrophilicity of the membranes tends to increase with the addition of inorganic components, which can be advantageous for cell adhesion.^15,67^ Likewise, it is likely that the wetting properties are also affected by the fiber diameter. Although WCA is similar between the hybrids, the membrane with a higher fiber diameter, 12–150–14, presents a slightly lower contact angle. As observed by Allo et al., two hybrids with different fiber diameters showed different times of spreading velocity of water droplets.^66^ The velocity of water droplets on the scaffold with coarser fibers was higher than on the one with the finer fibers.^66^ Hence, it is probable that the hybrid membrane with a reduced fiber diameter will not only exhibit a lower porosity but also exhibit a lower spreading velocity of water droplets compared with the membrane with a greater fiber diameter, as observed during the test.^66^ The denser structure obtained using a small fiber diameter may result in lower total pore volume and porosity.^66^

**Figure 10:**
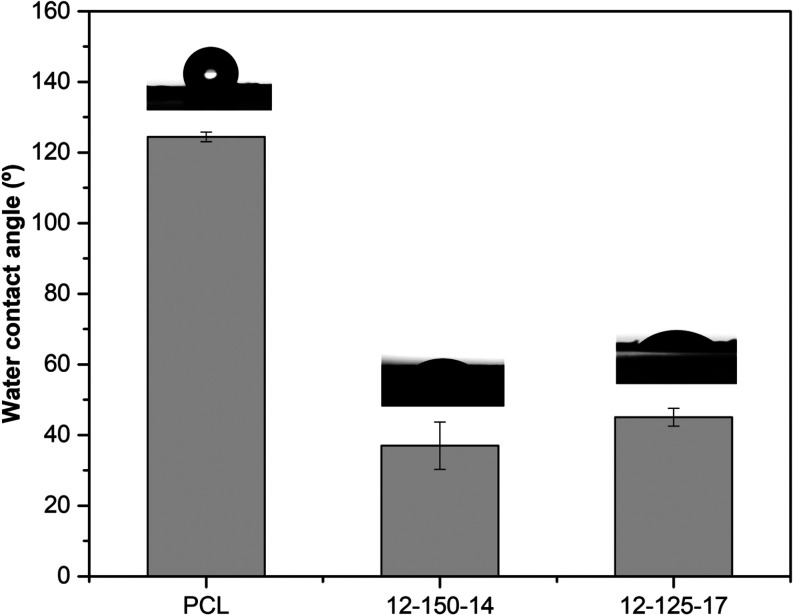
Water contact angle analysis of PCL, hybrid 12–150–14, and 12–125–17 nanofiber membranes.

### 3.3 *In Vitro* Cellular Assay

PCL and O/I hybrid membranes were seeded with L929 fibroblasts, a standard cell line used for cytotoxicity studies^68^ and cultured for 1, 3, and 7 days. Cell viability/proliferation was evaluated by the Resazurin assay throughout the culture period, Figure 11(A). The three membranes allowed the attachment of viable cells, as is evident by the positive values observed at day 1. The hybrid membranes yielded slightly higher values compared with the PCL membrane, but the differences were not statistically significant. Following, all samples allowed cell proliferation through the 7 day culture time, with the growth rate being higher from day 3 to day 7. The O/I hybrid membranes presented higher values compared with the PCL sample. However, the 12–125–17 membrane allowed the highest cell proliferation with values being statistically different from those found in the O/I 12–150–14 and PCL samples (at days 3 and 7). In another cell viability/proliferation method, Figure 11(B) shows low-magnification images of 7-day fibroblast-colonized membranes stained by the MTT assay. The violet-blue-stained cell layer, denoting the viable cells, was more abundant in the hybrid 12–125–17 membrane, which is in line with that observed in the Resazurin assay, Figure 11(A). Samples were also observed by SEM and representative images of 7-day cultured samples are presented in Figure 11(C). Low-magnification images showed cells with elongated morphology on PCL and the hybrid 12–150–14 sample, and a more polygonal appearance on the 12–125–17 membrane. Further, this membrane displayed a more abundant and uniform cell layer compared with the other two samples. Cell-to-cell contact appeared to be limited over PCL, but intricate and complex, over the O/I membranes. High-magnification images evidenced an intimate cell interaction with the underlying fibrous structure of the hybrid membranes, contrasting with that observed on the PCL sample.

**Figure 11:**
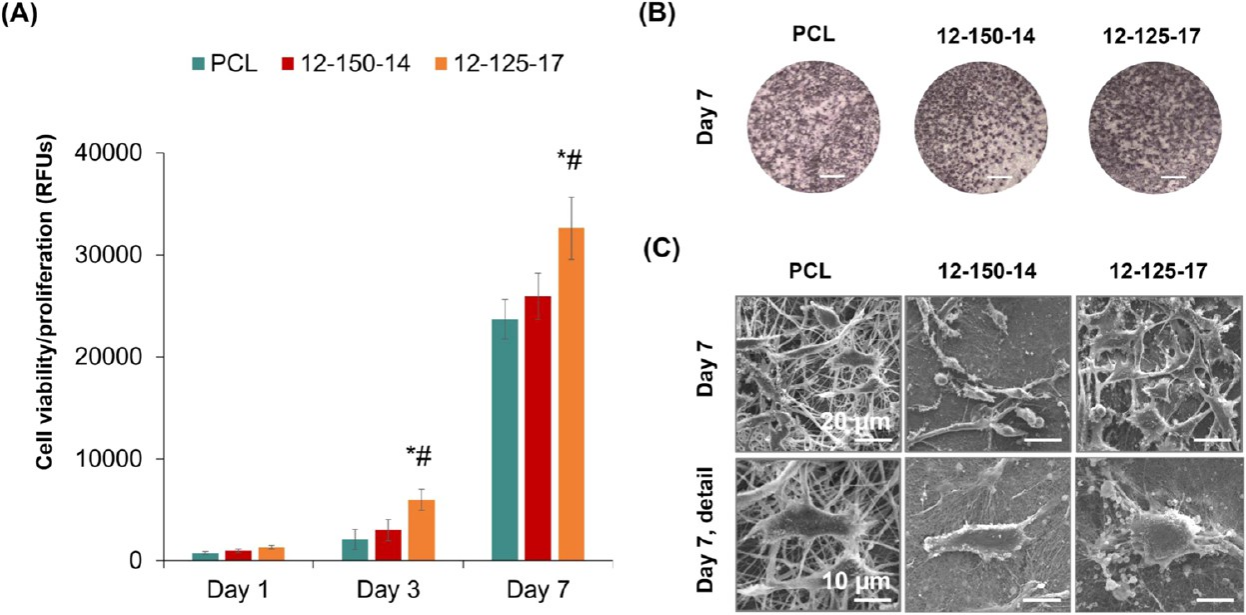
Cytocompatibility of PCL, 12–150–14, and 12–125–17 membranes seeded with L929 fibroblasts. Cell viability/proliferation of L929 cells cultured for 1, 3, and 7 days (A, Resazurin assay) and 7 days (B, MTT assay, scale bar = 25 mm). (C) SEM observation of colonized samples at day 7; scale bar = 20 μm (top line) and 10 μm (bottom line). A: significant difference from PCL (*) and hybrid 12–150–14 sample (#). RFUs: relative fluorescence units.

Compared with PCL, the results clearly showed a better performance of the hybrid membranes. Both were cytocompatible, allowing cell proliferation with the formation of an organized cell layer interacting with the underlying fibrous membrane. No signs of cytotoxicity were detected. The distinct physicochemical profiles of PCL and O/I membranes determine the differences in cell behavior. Relevant parameters include the surface hydrophobicity/hydrophilicity, surface area/fiber diameter, degradation rate, and *in vitro* bioactivity. In addition, for the hybrid membranes, the calcium and borate ions that are released during the culture time due to the membrane’s degradation may have a significant impact on the cell proliferation.^69^ Overall, *in vitro* available data showed that the effects are dose-dependent, but a suitable rate of ion release induced cell proliferation, supporting the applications of BG in wound healing.^70^ In the present work, the hybrid 12–125–17 membrane appears to present an optimal combination of surface parameters, leading to the best cell growth performance. This would include a hydrophilic surface, a high surface area linked to thinner fibers, an appropriate inorganic composition and degradation rate (Figure S3), and its *in vitro* bioactivity (Figures S4–S6).

## 4 Conclusions

In the present study, an organic–inorganic hybrid solution based on borosilicate and calcium compositions with PCL polymer phase was produced *via* *in situ* sol–gel process and homogeneous electrospun mats at the nanometer scale were achieved. A green solvent as acetic acid was used and demonstrated to be effective in the present system. This system showed to be spinnable and no defects, such as agglomeration of nanoparticles, were visible in the membranes. SEM results indicated that fiber diameter and morphology of electrospun mats can be controlled by appropriately manipulating ES parameters. Nonwoven and bead-free fibers were obtained for low flow rates (125–150 μL·h^–1^) and voltages (14–17 kV), particularly at 12 cm TCD. Compared with pure PCL solution, the hybrid solution had a more stable spinning, probably due to higher conductivity, and consequently, uniform and continuous fibers were produced. The incorporation of inorganic parts led to a significant decrease in the fiber’s diameter to approximately 100 nm. Furthermore, Si and Ca ions were homogeneously distributed through the nanofibers. From IR spectra, polymer and silica networks were linked by hydrogen bonds and NMR results confirmed the integration of boron in the network. The different electrospinning parameters tested on the hybrid solution affected the fiber diameter and, consequently, the mechanical properties, improving them as the fiber diameter decreased. Inorganic components enhanced the wettability properties of the polymeric membranes, being higher for coarse fiber. The hybrid materials were able to release boron and calcium ions from the structure and create a calcium-phosphate phase layer on the fiber’s surface, contrary to the neat PCL membrane. *In vitro* cellular assays showed an improvement of cell proliferation on O/I hybrids as well as cytocompatibility with better results for sample 12–125–17. The present study showed the potential of this green hybrid system for biomedical applications when processed as electrospun nanofiber membranes. Furthermore, this study reveals that process adjustments significantly affected mechanical properties, degradability, and cytocompatibility of the material, paving the way to further explore the biological properties of these hybrid membranes, namely, their angiogenesis ability.

## Supplementary Material



## Notes

Viscosity measurements (Figure S1); SEM micrographs of electrospun membranes obtained under different electrospinning conditions (Figure S2); experimental details and results of *in vitro* acellular assay: degradation (Figure S3) and bioactivity analysis, such as SEM micrographs (Figure S4), ATR-FTIR (Figure S5a–c), and XRD spectra (Figure S5d–f) of the samples after incubation in PBS; ionic release data assessed using ICP-OES technique (Figure S6) (PDF)

## Abbreviations

AA: acetic acid
ATR-FITR: attenuated total reflectance Fourier transform infrared spectroscopy
B: boron
BG: bioactive glass
Ca: calcium
CaAc: calcium acetate
CPD: critical point drying
DTG: derivate thermogravimetry
DSC: differential scanning calorimetry
E: elastic modulus, Young’s modulus
ES: electrospinning
EDS: energy-dispersive X-ray spectroscopy
ECM: extracellular matrix
FA: formic acid
HCl: hydrochloric acid
ICP-OES: inductively coupled plasma optimal emission spectroscopy
IPA: isopropyl alcohol
MAS: magical angle spinning
NMR: nuclear magnetic resonance
O/I: organic–inorganic
PBS: phosphate-buffered saline
PCL: poly­(ε-caprolactone)
RFUs: relative fluorescence units
RT: room temperature
SEM: scanning electron microscopy
STEM: scanning transmission electron microscopy
Si: silicon
TEOS: tetraethyl orthosilicate
TG: thermogravimetric
TCD: tip-to-collector distances
TMB: trimethyl borate
UV: ultraviolet
WCA: water contact angle

## References

[1] Bhardwaj N., Kundu S. C. (2010). Electrospinning: A Fascinating Fiber Fabrication Technique. Biotechnol. Adv..

[2] Keirouz A., Wang Z., Reddy V. S., Nagy Z. K., Vass P., Buzgo M., Ramakrishna S., Radacsi N. (2023). The History of Electrospinning:
Past, Present, and Future Developments. Adv.
Mater. Technol..

[3] Salas-Ambrosio P., Morales-Patlan E., Cedillo-Servin G., Tronnet A., Villavicencio K. P., Gómez-Lizárraga K., Benítez-Martínez J. A., Sanchez-Arevalo F. M., Velasquillo C., Ceapă C. D., Vera-Graziano R., Bonduelle C. (2024). Electrospinning Lysine-Polypeptide
Copolymers: Creating Microfiber Meshes for Biomedical Applications. ACS Appl. Polym. Mater..

[4] Allo B. A., Costa D. O., Dixon S. J., Mequanint K., Rizkalla A. S. (2012). Bioactive and Biodegradable Nanocomposites
and Hybrid
Biomaterials for Bone Regeneration. J. Funct.
Biomater..

[5] Sergi R., Cannillo V., Boccaccini A. R., Liverani L. (2020). Incorporation of Bioactive
Glasses Containing Mg, Sr, and Zn in Electrospun PCL Fibers by Using
Benign Solvents. Appl. Sci..

[6] Tabia Z., Akhtach S., Bricha M., El Mabrouk K. (2021). Tailoring
the Biodegradability and Bioactivity of Green-Electrospun Polycaprolactone
Fibers by Incorporation of Bioactive Glass Nanoparticles for Guided
Bone Regeneration. Eur. Polym. J..

[7] Gritsch L., Cédric Bossard A., Jallot E., Jones J. R., Lao J. (2023). Bioactive Glass-Based
Organic/Inorganic Hybrids: An Analysis of the
Current Trends in Polymer Design and Selection. J. Mater. Chem. B.

[8] Coelho S. A. R., Almeida J. C., Unalan I., Detsch R., Salvado I. M. M., Boccaccini A. R., Fernandes M. H. V. (2021). Cellular
Response to Sol-Gel Hybrid Materials Releasing Boron and Calcium Ions. ACS Biomater. Sci. Eng..

[9] Coelho S. A. R., Kniep J., Barroca N., Almeida J. C., Fernandes M. H. V. (2023). Nanofibrous
Hybrid Scaffolds Based on PCL-Borosilicate System by a Green Sol-Gel
Process. Mater. Today Chem..

[10] van
Der Schueren L., De Schoenmaker B., Kalaoglu Ö. I., De Clerck K. (2011). An Alternative Solvent System for the Steady State
Electrospinning of Polycaprolactone. Eur. Polym.
J..

[11] Allo B. A., Rizkalla A. S., Mequanint K. (2010). Synthesis
and Electrospinning of
ε-Polycaprolactone-Bioactive Glass Hybrid Biomaterials via a
Sol-Gel Process. Langmuir.

[12] Li W., Shi L., Zhang X., Liu K., Ullah I., Cheng P. (2018). Electrospinning
of Polycaprolactone Nanofibers Using H2O as Benign Additive in Polycaprolactone/Glacial
Acetic Acid Solution. J. Appl. Polym. Sci..

[13] Ekram B., Abdel-Hady B. M., El-Kady A. M., Amr S. M., Waley A. I., Guirguis O. W. (2017). Optimum
Parameters for the Production of Nano-Scale
Electrospun Polycaprolactone to Be Used as a Biomedical Material. Adv. Nat. Sci.: Nanosci. Nanotechnol..

[14] Liverani L., Boccardi E., Beltran A. M., Boccaccini A. R. (2017). Incorporation
of Calcium Containing Mesoporous (MCM-41-Type) Particles in Electrospun
PCL Fibers by Using Benign Solvents. Polymers.

[15] Gritsch L., Granel H., Charbonnel N., Jallot E., Wittrant Y., Forestier C., Lao J. (2022). Tailored Therapeutic Release from
Polycaprolactone-Silica Hybrids for the Treatment of Osteomyelitis:
Antibiotic Rifampicin and Osteogenic Silicates. Biomater. Sci..

[16] Almeida J. C., Wacha A., Gomes P. S., Fernandes M. H. R., Fernandes M. H. V., Salvado I. M. M. (2016). PDMS-SiO2-TiO2-CaO
Hybrid Materials - Cytocompatibility and Nanoscale Surface Features. Mater. Sci. Eng., C.

[17] Liverani L., Liguori A., Zezza P., Gualandi C., Toselli M., Boccaccini A. R., Focarete M. L. (2022). Nanocomposite Electrospun
Fibers
of Poly­(ε-Caprolactone)/Bioactive Glass with Shape Memory Properties. Bioact. Mater..

[18] Mondal D., Rizkalla A. S., Mequanint K. (2016). Bioactive
Borophosphosilicate-Polycaprolactone
Hybrid Biomaterials: Via a Non-Aqueous Sol Gel Process. RSC Adv..

[19] Shin K. H., Koh Y. H., Kim H. E. (2013). Synthesis
and Characterization of
Drug-Loaded Poly­(ε -Caprolactone)/Silica Hybrid Nanofibrous
Scaffolds. J. Nanomater..

[20] Lepry W. C., Smith S., Liverani L., Boccaccini A. R., Nazhat S. N. (2016). Acellular Bioactivity of Sol-Gel
Derived Borate Glass-Polycaprolactone
Electrospun Scaffolds. Biomed. Glasses.

[21] Lepry W. C., Nazhat S. N. (2015). Highly Bioactive Sol-Gel-Derived
Borate Glasses. Chem. Mater..

[22] Sakai S., Kawakami K., Taya M. (2012). Controlling
the Diameters of Silica
Nanofibers Obtained by Sol-Gel/Electrospinning Methods. J. Chem. Eng. Jpn..

[23] Van
Der Schueren L., Steyaert I., De Schoenmaker B., De Clerck K. (2012). Polycaprolactone/Chitosan Blend Nanofibres Electrospun
from an Acetic Acid/Formic Acid Solvent System. Carbohydr. Polym..

[24] Liverani L., Boccaccini A. R. (2016). Versatile
Production of Poly­(Epsilon-Caprolactone)
Fibers by Electrospinning Using Benign Solvents. Nanomaterials.

[25] Zong X., Kim K., Fang D., Ran S., Hsiao B. S., Chu B. (2002). Structure
and Process Relationship of Electrospun Bioabsorbable Nanofiber Membranes. Polymer.

[26] Catauro M., Raucci M. G., De Marco D., Ambrosio L. (2006). Release Kinetics of
Ampicillin, Characterization and Bioactivity of TiO 2 /PCL Hybrid
Materials Synthesized by Sol-Gel Processing. J. Biomed. Mater. Res. A.

[27] Ding Y., Roether J. A., Boccaccini A. R., Schubert D. W. (2014). Fabrication of Electrospun
Poly (3-Hydroxybutyrate)/Poly (ε-Caprolactone)/Silica Hybrid
Fibermats with and without Calcium Addition. Eur. Polym. J..

[28] Allo B. A., Rizkalla A. S., Mequanint K. (2010). Synthesis
and Electrospinning of
ε-Polycaprolactone-Bioactive Glass Hybrid Biomaterials via a
Sol-Gel Process. Langmuir.

[29] Nie K., Zheng S., Lu F., Zhu Q. (2005). Inorganic-Organic Hybrids
Involving Poly­(ε-Caprolactone) and Silica Network: Hydrogen-Bonding
Interactions and Isothermal Crystallization Kinetics. J. Polym. Sci., Part B:Polym. Phys..

[30] Ding Y., Roether J. A., Boccaccini A. R., Schubert D. W. (2014). Fabrication of Electrospun
Poly (3-Hydroxybutyrate)/Poly (ε-Caprolactone)/Silica Hybrid
Fibermats with and without Calcium Addition. Eur. Polym. J..

[31] Aguiar H., Serra J., González P., León B., Gonzalez P., Leon B. (2009). Structural Study of
Sol-Gel Silicate
Glasses by IR and Raman Spectroscopies. J. Non
Cryst. Solids.

[32] Choi S.-S., Goo Lee S., Joo Y. L. (2003). Silica Nanofibers from
Electrospinning/Sol-Gel Process. J. Mater. Sci.
Lett..

[33] Peña-Alonso R., Rubio J., Rubio F., Oteo J. (2002). A FT-IR Study of the
Synthesis of Boron Ormosils by Means of the Sol-Gel Process. J. Sol-Gel Sci. Technol..

[34] Peña-Alonso R., Sorarù G. D. (2007). Synthesis
and Characterization of Hybrid Borosiloxane
Gels as Precursors for Si-B-O-C Fibers. J. Sol-Gel
Sci. Technol..

[35] Rathinavel S., Korrapati P. S., Kalaiselvi P., Dharmalingam S. (2021). Mesoporous
Silica Incorporated PCL/Curcumin Nanofiber for Wound Healing Application. Eur. J. Pharm. Sci..

[36] Peña-Alonso R., Rubio J., Rubio F., Oteo J. L. (2003). FT-IR and
Porosity
Study of Si-B-C-O Materials Obtained from TEOS-TEB-PDMS Derived Gel
Precursors. J. Sol-Gel Sci. Technol..

[37] Almeida J. C., Castro A. G. B., Salvado I. M. M., Margaça F. M. A., Vaz Fernandes M. H. (2014). A New Approach to the Preparation
of PDMS–SiO2 Based Hybrids – A Structural Study. Mater. Lett..

[38] Almeida J. C., Wacha A., Gomes P. S., Alves L. C., Fernandes M. H. V., Salvado I. M. M., Fernandes M. H. R. (2016). A Biocompatible
Hybrid Material with Simultaneous Calcium and Strontium Release Capability
for Bone Tissue Repair. Mater. Sci. Eng., C.

[39] Grandi S., Tomasi C., Cassinelli V., Cucca L., Profumo A., Mustarelli P., Balduini C. (2012). SiO2-B2O3 Xerogels: The Problem of
Boron Leaching. J. Non Cryst. Solids.

[40] Beckett M. A., Rugen-Hankey M. P., Varma K. S. (2000). Trimethoxyboroxine as an “oxygen-Transfer”
Reagent: A Non-Aqueous “Sol-Gel” Route to Alkali-Free
Borosilicate Glass. Chem. Commun..

[41] Oliveira J. E., Mattoso L. H. C., Orts W. J., Medeiros E. S. (2013). Structural and Morphological
Characterization of Micro and Nanofibers Produced by Electrospinning
and Solution Blow Spinning: A Comparative Study. Adv. Mater. Sci. Eng..

[42] Bölgen N., Menceloğlu Y. Z., Acatay K., Vargel I., Pişkin E. (2005). In Vitro and
in Vivo Degradation of Non-Woven Materials Made of Poly­(ε-Caprolactone)
Nanofibers Prepared by Electrospinning under Different Conditions. J. Biomater. Sci. Polym. Ed..

[43] Almeida J. C., Wacha A., Bóta A. B., Alm Asy L., Helena M., Fernandes V., Margaça F. M.
A., Salvado I. M. M. (2015). PDMS-SiO2
Hybrid Materials: A New Insight into the Role of Ti and Zr as Additives. Polymer.

[44] Lana S. L. B., Seddon A. B. (1998). X-Ray Diffraction
Studies of Sol-Gel Derived Ormosils
Based on Combinations of Tetramethoxysilane and Trimethoxysilane. J. Sol-Gel Sci. Technol..

[45] Sorarù G. D., Dallabona N., Gervais C., Babonneau F. (1999). Organically
Modified SiO2-B2O3 Gels Displaying a High Content of Borosiloxane
(= B-O-Si) Bonds. Chem. Mater..

[46] Zeng W., Cheng N.-m., Liang X., Hu H., Luo F., Jin J., Li Y.-w. (2022). Electrospun Polycaprolactone
Nanofibrous Membranes
Loaded with Baicalin for Antibacterial Wound Dressing. Sci. Rep..

[47] Hernández A. R., Contreras O. C., Acevedo J. C., Guadalupe L., Moreno N. (2013). Poly­(ε-Caprolactone) Degradation under Acidic
and Alkaline Conditions. Am. J. Polym. Sci..

[48] Balogh Z., Len A., Baksa V., Krajnc A., Herman P., Szemán-Nagy G., Czigány Z., Fábián I., Kalmár J., Dudás Z. (2024). Nanoscale Structural Characteristics and in Vitro Bioactivity
of Borosilicate–Poly­(Vinyl Alcohol) (PVA) Hybrid Aerogels for
Bone Regeneration. ACS Appl. Nano Mater..

[49] Brus J. (2002). Solid-State
NMR Study of Phase Separation and Order of Water Molecules and Silanol
Groups in Polysiloxane Networks. J. Sol-Gel
Sci. Technol..

[50] Leonova E., Izquierdo-Barba I., Arcos D., López-Noriega A., Hedin N., Vallet-Regí M., Edén M. (2008). Multinuclear
Solid-State NMR Studies of Ordered Mesoporous Bioactive Glasses. J. Phys. Chem. C.

[51] Wu J., Stebbins J. F. (2014). Cation Field Strength
Effects on Boron Coordination
in Binary Borate Glasses. J. Am. Ceram. Soc..

[52] Morin E. I., Wu J., Stebbins J. F. (2014). Modifier
Cation (Ba, Ca, La, Y) Field Strength Effects
on Aluminum and Boron Coordination in Aluminoborosilicate Glasses:
The Roles of Fictive Temperature and Boron Content. Appl. Phys. A.

[53] Du L.-S., Stebbins J. F. (2003). Nature of Silicon–Boron
Mixing in Sodium Borosilicate
Glasses: A High-Resolution ^11^ B and ^17^ O NMR
Study. J. Phys. Chem. B.

[54] Soraru G. D., Babonneau F., Gervais C., Dallabona N. (2000). Hybrid RSiO1.5/B2O3
Gels from Modified Silicon Alkoxides and Boric Acid. J. Sol-Gel Sci. Technol..

[55] Peña-Alonso R., Tamayo A., Rubio F., Rubio J. (2005). Influence of Boron
Concentration on the Surface Properties of TEOS-PDMS Hybrid Materials. J. Sol-Gel Sci. Technol..

[56] Irwin A. D., Holmgren J. S., Zerda T. W., Jonas J. (1987). Spectroscopic Investigations
of Borosiloxane Bond Formation in the Sol-Gel Process. J. Non Cryst. Solids.

[57] Irwin A. D., Holmgren J. S., Jonas J. (1988). Solid State
29Si and 11B NMR Studies
of Sol-Gel Derived Borosilicates. J. Non-Cryst.
Solids.

[58] Touati F., Sediri F., Gharbi N. (2009). Preparation and Characterization
of Mesoporous Lithium Borosilicate Material via the Sol-Gel Process. Mater. Sci. Eng., C.

[59] Prajongtat P., Sriprachuabwong C., Wongkanya R., Dechtrirat D., Sudchanham J., Srisamran N., Sangthong W., Chuysinuan P., Tuantranont A., Hannongbua S., Chattham N. (2019). Moisture-Resistant
Electrospun Polymer Membranes for
Efficient and Stable Fully Printable Perovskite Solar Cells Prepared
in Humid Air. ACS Appl. Mater. Interfaces.

[60] Lee E. J., Teng S. H., Jang T. S., Wang P., Yook S. W., Kim H. E., Koh Y. H. (2010). Nanostructured Poly­(ε-Caprolactone)-Silica
Xerogel Fibrous Membrane for Guided Bone Regeneration. Acta Biomater..

[61] Cipitria A., Skelton A., Dargaville T. R., Dalton P. D., Hutmacher D. W. (2011). Design,
Fabrication and Characterization of PCL Electrospun Scaffolds - A
Review. J. Mater. Chem..

[62] Meka S. R. K., Verma S. K., Agarwal V., Chatterjee K. (2018). In Situ Silication
of Polymer Nanofibers to Engineer Multi-Biofunctional Composites. ChemistrySelect.

[63] Eglin D., Perry C. C., Ali S. A. M. (2005). A New
Class II Poly (ε-Caprolactone)-Silica
Hybrid: Synthesis and in Vitro Apatite Forming Ability. J. Bioact. Compat. Polym..

[64] Castro A. G. B., Diba M., Kersten M., Jansen J. A., van den
Beucken J. J. J. P., Yang F. (2018). Development of a PCL-Silica Nanoparticles
Composite Membrane for Guided Bone Regeneration. Mater. Sci. Eng.,C.

[65] Ezhilarasu H., Ramalingam R., Dhand C., Lakshminarayanan R., Sadiq A., Gandhimathi C., Ramakrishna S., Bay B. H., Venugopal J. R., Srinivasan D. K. (2019). Biocompatible
aloe vera and tetracycline hydrochloride loaded hybrid nanofibrous
scaffolds for skin tissue engineering. Int.
J. Mol. Sci..

[66] Allo B. A., Lin S., Mequanint K., Rizkalla A. S. (2013). Role of Bioactive 3D Hybrid Fibrous
Scaffolds on Mechanical Behavior and Spatiotemporal Osteoblast Gene
Expression. ACS Appl. Mater. Interfaces.

[67] Bossard C., Granel H., Wittrant Y., Jallot É., Lao J., Vial C., Tiainen H. (2018). Polycaprolactone/Bioactive
Glass
Hybrid Scaffolds for Bone Regeneration. Biomed.
Glasses.

[68] International Organization for Standardization . ISO 10993–5 Biological Evaluation of Medical Devices - Part 5: Tests for in Vitro Cytotoxicity; International Standard, 2009.

[69] Shafaghi R., Rodriguez O., Wren A. W., Chiu L., Schemitsch E. H., Zalzal P., Waldman S. D., Papini M., Towler M. R. (2021). In Vitro
Evaluation of Novel Titania-Containing Borate Bioactive Glass Scaffolds. J. Biomed. Mater. Res..

[70] Ege D., Zheng K., Boccaccini A. R. (2022). Borate
Bioactive Glasses (BBG): Bone
Regeneration, Wound Healing Applications, and Future Directions. ACS Appl. Bio Mater..

