# Supplementary material for: Organic–Inorganic Hybrid Nanofiber Membranes by Electrospinning: Engineering Features and Cytocompatibility
Source: ACS Appl Polym Mater. 2025 Jul 11;7(14):9010–24. doi: 10.1021/acsapm.5c01072 (PMC12821186; doi:10.1021/acsapm.5c01072)
Supplement: Supplementary file 1 [file ap5c01072_si_001.pdf]

Supporting Information

Organic-inorganic hybrid nanofibers membranes by electrospinning: engineering features and cytocompatibility

Soraia A.R. Coelho [(1)], *Liliana Grenho* [(2)], *Maria Helena Raposo Fernandes* [(2)], *Maria Helena Vaz Fernandes* [(1)], *José Carlos Almeida* [(1)*]

[(1)] CICECO – Materials Institute of Aveiro, Department of Materials and Ceramic Engineering, University of Aveiro, 3810-193 Aveiro, Portugal

[(2)] LAQV/REQUIMTE, Faculty of Dental Medicine, University of Porto, Rua Dr. Manuel Pereira da Silva, 4200-393 Porto, Portugal

*** Corresponding author.**

J. Carlos Almeida

E-mail: jcalmeida@ua.pt

1. Materials and methods

1.1. Rheology

The change of viscosity by time (η) was calculated by using the following equation (S1):

$$\eta = 100 - \left(\frac{\eta - \eta_0}{\eta_0}\right) \times 100$$

(S1)

where the η is the viscosity after the determined time and η_0 is the initial viscosity of the solution[1].

1.2. *In vitro* degradation and bioactivity study

The stability of the membranes was examined through weight loss and pH variation in phosphate-buffered saline (PBS), pH 7.4, during a period of 14 days. Square samples of 1 x 1 cm^2 (n=3) were immersed in the medium, in a ratio of 1:500 mass/volume, and at 37 ˚C in a shaking incubator at a constant speed of 100 rpm, without PBS refreshing. The samples were collected at predetermined timepoints: 1, 3, 6, 12 hours and 1, 3, 7, 14 days. At each selected time, the samples were withdrawn from the incubation medium, dried 24 hours in oven at 37 °C and weighed. The degradation was expressed as percentage of weight loss, calculated using the equation (S2):

$$Weight\ loss\ (\%) = \frac{W_0 - W}{W_0} \times 100$$

(S2)

where W_0 is the initial sample's weight, and $W$is the sample's measured weight after incubation in the degradation medium. The average of three measurements ± standard deviation was to use to express the results.

The bioactivity of the material *in vitro* was evaluated for all time points. After each period, the samples were collected and the concentrations of silicon, calcium and boron elements released from the membranes in the medium were evaluated, as well as phosphor ions. Inductively coupled plasma optical emission spectroscopy (ICP-OES, Jobin Yvon Activa M, France) was used to monitor the elements released. The microstructure and structure of the samples after immersing in PBS were analyzed by SEM-EDS, ATR-FTR and X-ray diffraction (XRD) techniques to confirm the presence of a calcium-phosphate phase, an indicator of bioactivity. For XRD measurements, the equipment used was X'Pert PRO diffractometer (Malvern PANanlytical, Worcestershire, United Kingdom) equipped with a copper anode (Cu), which operates with a current of 40 mA and a voltage of 45 kV, emitting a radiation Kα1 of λ=1.540 Å and Kα2 of λ=1.544 Å. The spectra were recorded in the 2θ range 3-40°, with a step size of 0.0263°.

2. Results and discussion

2.1. Solution viscosity

Figure S1 presents the (a) viscosity and (b) viscosity change of the three solutions along time: PCL, hybrid and PCL-HCl solutions. The viscosity values were taken at 1000 s^{-1} shear rate. Observing the graphs (**Figure S1 a**), the hybrid and the PCL with the acid (PCL-HCl) showed lower viscosity values

compared with the organic solution. In terms of viscosity change (**Figure S1 b**), the PCL-HCl and hybrid solutions showed the same trend, a decay through time. Thus, the introduction of acid contributed to the decrease in the solution viscosity, probably due to scission of PCL chains that lowered the polymer molecular weight [2].

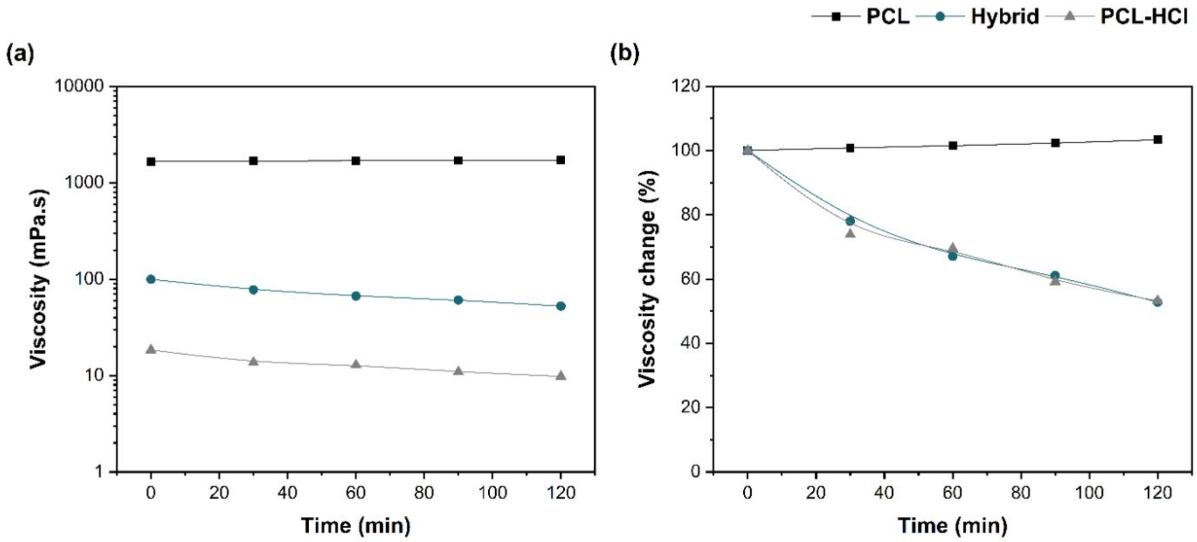

Figure S1. (a) viscosity and (b) viscosity change over time of the three solutions: PCL, hybrid and PCL-HCl (with the acid) solutions.

2.2. Characterization of electrospun membranes

2.2.1. Morphology analysis

Increasing the distance to 15 cm no improvements were observed on the membrane's morphology. **Figure S2** shows some representative SEM images of the membranes electrospun at 15 cm of TCD, for 125 (a) and 200 μL.h^{-1} (b), keeping constant the voltage (15-125-17 and 15-200-17 samples). Analyzing

the images, the resultant structure is mainly composed of droplets and particles, thus non-uniform meshes were produced, probably due to the instability of the jet and spraying that occurred during the electrospinning of the solution. For higher distances, as TCD 18 cm (**Figure S2 c, d**) better homogeneity of the membranes was obtained for low flow rates and voltages, between 125-150.μL h^{-1} and 14-17 kV, as can be seen in **Figure S2 (c)** and **(d)**. When the flow rate was above 150 μL.h^{-1}, higher voltages were probably required, since spraying started to occur using low voltages and, consequently, no fibrous material was formed, only droplets on the aluminum foil. It is the case of 18-200-17 sample, as observed in **Figure S2 (d)**.

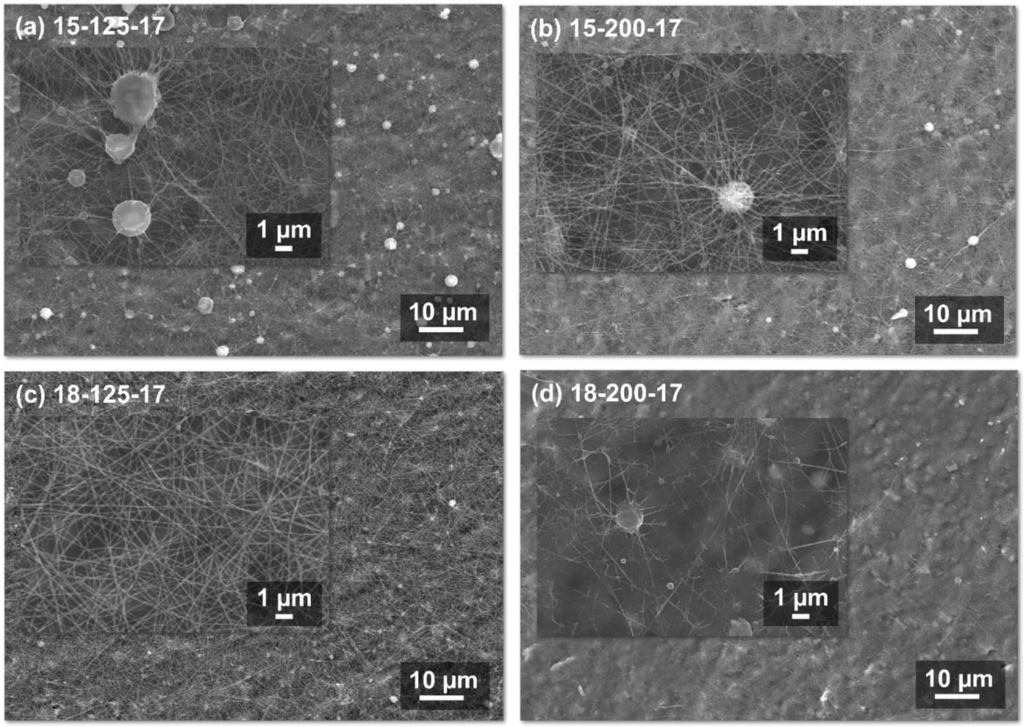

Figure S2. SEM micrographs of hybrid electrospun membranes, at x1.0k and x6.0k magnification, produced at TCD of 15 cm (a, b) and 18 cm (c, d), for 125 $\mu L.h^{-1}$ (a, c) and 200 (b, d) $\mu L.h^{-1}$ of flow rate, keeping constant the voltage (17 kV).

2.2.2. *In vitro* acellular assay: degradation study

The *in vitro* degradation assay was conducted by determining the weight of the membrane after exposure to PBS for a period of 14 days. The percentage of weight loss is shown in **Figure S3 (a)**. The PCL membrane exhibited a maximum weight loss of 8.4% after 3 days of contact with the solution. This weight loss can be explained by the hydrolytic degradation process that creates hydroxyl and carboxyl groups via breaking down the long polymer chains of PCL [3,4]. PCL membrane showed a minimal weight loss (less than 10%) after 14 days of incubation. This slow degradation is due to its hydrophobic nature, as confirmed by contact angle measurements [3,5,6]. The hydrophobic -CH_2 groups on PCL could delay water penetration and de-esterification [3,7,8]. Though, minor weight loss may occur as the amorphous parts break down more quickly than the crystalline areas, where polymer chains are densely packed [3]. Over time, PCL's hydrophilicity tends to increase, resulting in higher water absorption and, consequently, accelerating the PCL nanofiber breakdown [4].

The O/I hybrid nanofibers (12-125-17 and 12-150-14 samples) demonstrated distinct *in vitro* degradation profiles compared to the PCL membrane. Both hybrids began degrading at early stage of the assay (within 1 hour), with the 12-150-14 and 12-125-17 samples losing respectively 2.2% and 26.6% of mass. A rapid initial weight loss occurred in 12-125-17 membrane during the first 12 hours, followed by

a slower degradation. Conversely, 12-150-14 sample showed a slow degradation, slightly higher than PCL membrane. These results align with the ones reported by Bossard *et al.*, for class I polycaprolactone-based hybrids, where the preliminary weight loss is due to the dissolution of inorganic components, followed by PCL de-esterification, resulting in a weight loss of 15-27 % over 14 days [7,9]. Hence, the early stage of hybrid degradation involved the dissolution of inorganic components, such as therapeutic ions (Ca^{2+} and B^{3+}), in the surface of hybrid fibers into the medium. The presence of these ions can induce PCL degradation by acting as nucleation sites for hydrolytic attack, which can be explained by the higher hydrophilicity of hybrid membranes. This facilitated water absorption and accelerated the hydrolytic chain scission of ester bonds. Moreover, knowing that acid and basic conditions can accelerate PCL degradation, herein inorganic ions created an acidic environment that promoted PCL degradation, as noted by Tabia *et al.* and Bossard *et al.* in basic conditions [3,7]. The degradation rates of hybrid samples depend on fiber diameter. The slightly thicker 12-150-14 fibers showed a more uniform and slower degradation behavior compared to the thinner 12-125-17 fibers, which degraded more rapidly probably owing to a higher surface-to-volume ratio [10]. Generally, hybrids degrade faster than PCL alone, indicating that the addition of inorganic components like silicon, boron, and calcium ions enhances PCL degradation. Similar results were also found in other studies [3,7,11].

Changes in pH were also monitored, with the values depicted in Figure S3 (b). PCL maintained a relatively stable pH, decreasing slightly to 7.26 over time. However, the hybrids showed a more significant drop, with 12-125-17 exhibiting, in the most part of time, the lowest pH values. This agrees with mass degradation data, where 12-125-17 membrane presented a slightly higher degradation rate, thus having higher effect on the pH of the medium. The reduction in the pH could be attributed to the cleavage of PCL

ester bonds by water, causing the formation of carboxyl end-groups that dissolve and further catalyze degradation [10].

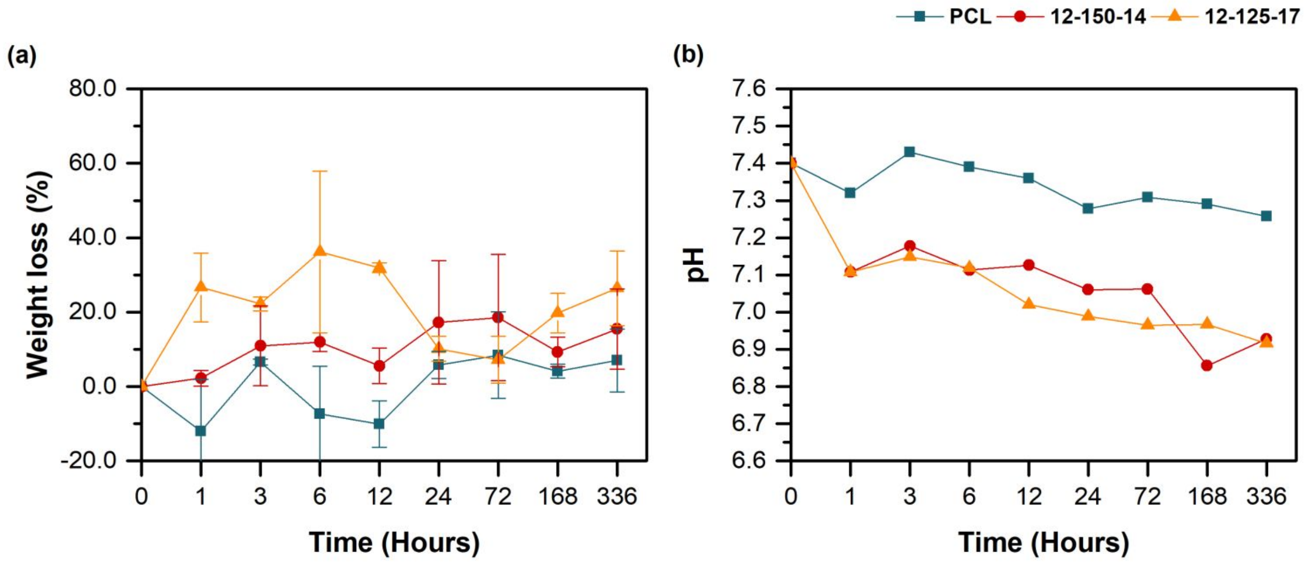

Figure S3. In vitro study: (a) weight loss and (b) pH of the medium as a function of immersion time.

2.2.3. *In vitro* acellular assay: bioactivity study

Figure S4 the SEM micrographs and EDS elemental analysis of PCL and O/I hybrids membranes before (hour 0) and after incubation in PBS for 1 hour, 24 hours and 168 hours (7 days), at x15.0k magnification. After 1 hour, the hybrid samples were already covered with heterogenous and spherically shaped particles. The same was observed for other incubation times. The cauliflower shaped structures in hybrid micrographs may suggest a calcium-phosphate layer, which was confirmed by EDS analysis with the identification of the phosphorus (P) peak in the spectrum of the hybrids. Conversely, no apatite layer deposition was detected on the PCL membrane after 1 hour, 24 hours and 168 hours. This occurred in all

time points up to 336 hours (14 days) of incubation. EDS analysis of the PCL membrane demonstrated that no calcium or phosphorus elements were detected.

The presence of an amorphous phase of calcium-phosphate (Ca-P) layer or hydroxyapatite (HA) crystals on the PCL and O/I hybrid samples was investigated by ATR-FTIR spectroscopy and X-ray. **Figure S5** presents the IR (a, b, c) and XRD (d, e, f) spectra obtained for each sample before (0 h) and after immersion for 1 h, 24 h and 168 h in PBS. After 1 hour of incubation, the peaks at *ca.* 560 cm^{-1} and 602 cm^{-1} were detected in both hybrids, being characteristic of crystalline P-O vibrational band [12,13]. Along the incubation time, this double band is still present. Other band at *ca.* 1026 cm^{-1} appeared in 12-150-14 and 12-125-17 hybrid samples, corresponding to P-O stretching vibration of PO_4^{3-}, being more distinct with the time of incubation [13]. Conversely, in PCL samples these peaks were not observed, hence no calcium-phosphate layer was formed in the PCL control membranes, as noticed previously in SEM-EDS analysis. **Figure S5 (d-f)** show the XRD pattern acquired from (d) PCL, (e) hybrids 12-150-14 and (f) 12-125-17. The appearance of peaks at ~32 ° confirmed that HA phase had been deposited on the membrane's surface [14,15]. The intensity of this peak is almost similar for both hybrids, but after 168 h of incubation a strong hydroxyapatite peak was observed. The ATR-FTIR and XRD results confirmed that the hybrid samples were bioactive, and agree with the SEM micrographs, where a dense layer of HA is confirmed.

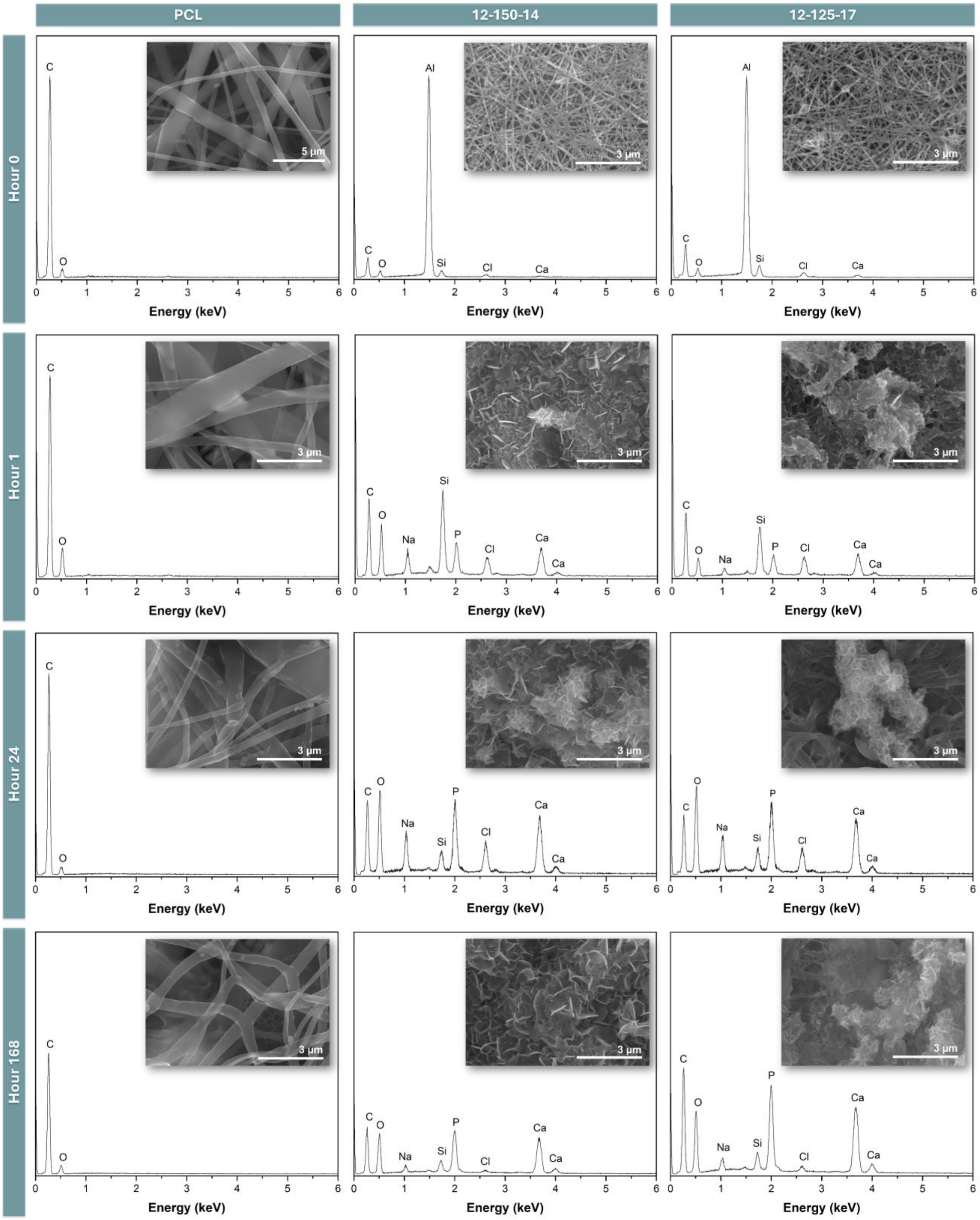

PCL
12-150-14
12-125-17
Hour 0
Hour 1
Hour 24
Hour 168
Energy (keV)
C
O
Al
Si
Cl
Ca
Na
P
5 μm
3 μm

Figure S4. SEM micrographs, at x15.0k magnification, and respective EDS elemental analysis of PCL, 12-150-14 and 12-125-17 hybrid membranes before (hour 0) and after immersion for 1 hour, 24 hours and 168 hours in PBS. Scale bar of 5 μm for PCL at 0h, and 3 μm for the rest of the SEM images.

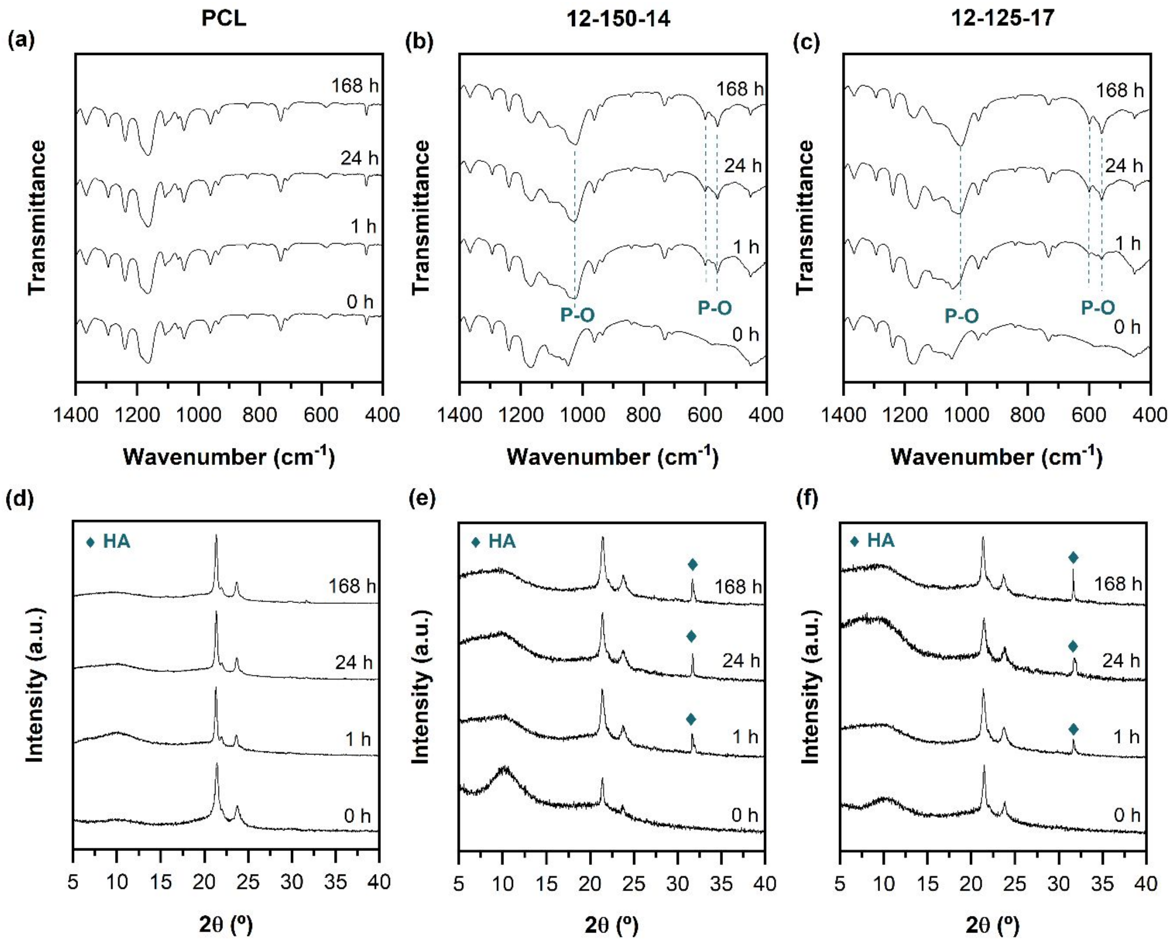

Figure S5. ATR-FTIR spectra of (a) PCL, (b) 12-150-14 and (c) 12-125-17 samples and XRD diffractograms of (d) PCL, (e) 12-150-14 and (f) 12-125-17 samples, before (0 h) and after incubation in PBS FOR 1 h, 24 h and 168 h.

Ion's release

The release of ionic boron (B), calcium (Ca), phosphorus (P) and silicon (Si) from the hybrid membranes was measured by the ICP-OES technique, as shown in **Figure S6.** Observing the B levels, a first release of boron was observed in the initial hours of immersion in both membranes. The 12-150-14 hybrid presented a more controlled and moderate release of B compared to the 12-125-17 hybrid sample, during the entire immersion period. The membrane with lower diameter showed a higher release of boron, increasing with time. The maximum release observed was 942 $\mu g.L^{-1}$ at 14 days, while for sample 12-150-14 the maximum was 450 $\mu g.L^{-1}$ at 7 days. According to some authors, during drug delivery via nanofibers, an increased drug release is observed for "*scaffolds with smaller fiber diameters compared to those with larger diameters, since the drug could not diffuse faster from the interior of the larger diameter nanofibers*" [16,17]. Besides, fibers with lower diameter presents higher specific surface area, which induces higher release of drugs [16]. As expected, the phosphorus concentration in the medium decreased in the first hours, reaching a controlled decrease after 3 days. This supports earlier findings that a Ca-P phase forms on the surface of the hybrid. Regarding calcium, a release of Ca ions was observed in the first hour as a result of the presence of this element in the hybrid structure. Though, immediately afterwards a decrease occurred up to 3 and 7 days, 12-150-14 and 12-125-17, respectively, probably due to the deposition of Ca-P phase. In Si ions, its concentration in the medium quickly increases in the first 24 hours, reaching a saturation plateau of *ca.* 45 ppm. This tendency has already been reported in hybrid materials and silica-derived sol-gel materials, attributed to the diffusion of unreacted components and silicate oligomers released from the hybrid matrix into the water [7,9,18,19]. These findings support the premise that PCL-silica hybrids can facilitate the gradual release of silicates, creating a suitable environment for cell's activity, such as adhesion, proliferation and differentiation [9].

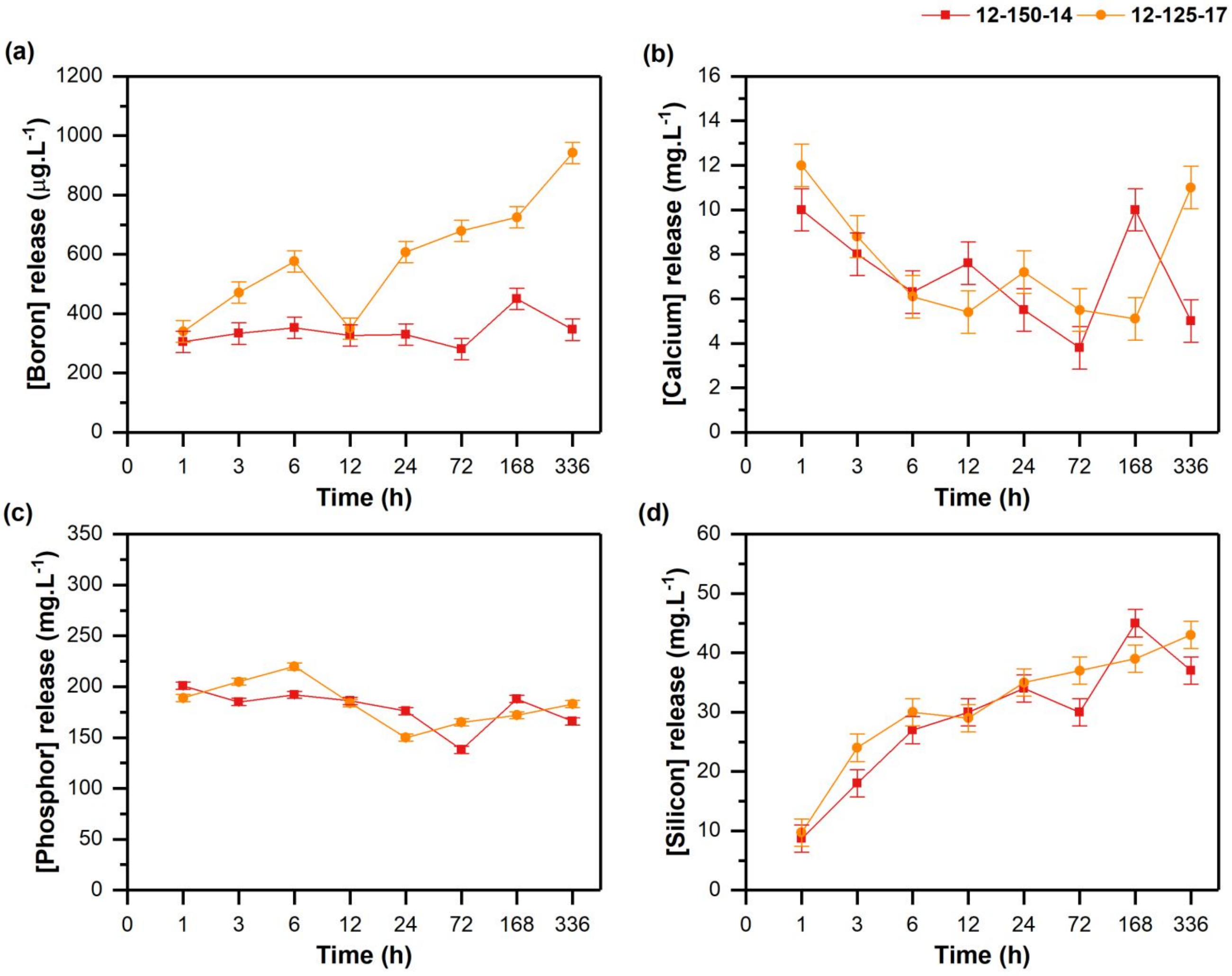

Figure S6. Release of (a) boron, (b) calcium, (c) phosphor, and (d) silicon from the 12-150-14 and 12-125-17 hybrid membranes after immersed in PBS for 14 days.

References

(1) Ekram, B.; Abdel-Hady, B. M.; El-Kady, A. M.; Amr, S. M.; Waley, A. I.; Guirguis, O. W. Optimum Parameters for the Production of Nano-Scale Electrospun Polycaprolactone to Be Used as a Biomedical Material. *Advances in Natural Sciences: Nanoscience and Nanotechnology* **2017**, *8* (4), 045018. https://doi.org/10.1088/2043-6254/aa92b4.

(2) Anaya-Mancipe, J. M.; de Figueiredo, A. C.; Rabello, L. G.; Dias, M. L.; da Silva Moreira Thiré, R. M. Evaluation of the Polycaprolactone Hydrolytic Degradation in Acid Solvent and Its Influence on the Electrospinning Process. *J Appl Polym Sci* **2024**, *141* (29). https://doi.org/10.1002/app.55662.

(3) Tabia, Z.; Akhtach, S.; Bricha, M.; El Mabrouk, K. Tailoring the Biodegradability and Bioactivity of Green-Electrospun Polycaprolactone Fibers by Incorporation of Bioactive Glass Nanoparticles for Guided Bone Regeneration. *Eur Polym J* **2021**, *161*, 110841. https://doi.org/10.1016/J.EURPOLYMJ.2021.110841.

(4) Henrique Lima, T.; Fernandes-Cunha, G. M.; Jensen, C. E. D. M.; Oréfice, R. L.; Junior, A. D. S. C.; Zhao, M.; Behar-Cohen, F.; Da Silva, G. R. Bioactive Glass Nanoparticles-Loaded Poly(□-Caprolactone) Nanofiber as Substrate for ARPE-19 Cells. *J Nanomater* **2016**, *2016*. https://doi.org/10.1155/2016/4360659.

(5) Ding, Y.; Li, W.; Correia, A.; Yang, Y.; Zheng, K.; Liu, D.; Schubert, D. W.; Boccaccini, A. R.; Santos, H. A.; Roether, J. A. Electrospun Polyhydroxybutyrate/Poly(□-Caprolactone)/Sol-Gel-Derived Silica Hybrid Scaffolds with Drug Releasing Function for Bone Tissue Engineering Applications. *ACS Appl Mater Interfaces* **2018**, *10* (17), 14540–14548. https://doi.org/10.1021/acsami.8b02656.

(6) Lee, E. J.; Teng, S. H.; Jang, T. S.; Wang, P.; Yook, S. W.; Kim, H. E.; Koh, Y. H. Nanostructured Poly(ε-Caprolactone)-Silica Xerogel Fibrous Membrane for Guided Bone Regeneration. *Acta Biomater* **2010**, *6* (9), 3557–3565. https://doi.org/10.1016/j.actbio.2010.03.022.

(7) Bossard, C.; Granel, H.; Wittrant, Y.; Jallot, É.; Lao, J.; Vial, C.; Tiainen, H. Polycaprolactone/Bioactive Glass Hybrid Scaffolds for Bone Regeneration. *Biomedical Glasses* **2018**, *4* (1), 108–122. https://doi.org/10.1515/bglass-2018-0010.

(8) Suwantong, O. Biomedical Applications of Electrospun Polycaprolactone Fiber Mats. *Polymers for Advanced Technologies*. John Wiley and Sons Ltd October 1, 2016, pp 1264–1273. https://doi.org/10.1002/pat.3876.

(9) Gritsch, L.; Granel, H.; Charbonnel, N.; Jallot, E.; Wittrant, Y.; Forestier, C.; Lao, J. Tailored Therapeutic Release from Polycaprolactone-Silica Hybrids for the Treatment of Osteomyelitis:

Antibiotic Rifampicin and Osteogenic Silicates. *Biomater Sci* **2022**, *10* (8), 1936–1951. https://doi.org/10.1039/d1bm02015c.

(10) Bölgen, N.; Menceloğlu, Y. Z.; Acatay, K.; Vargel, I.; Pişkin, E. In Vitro and in Vivo Degradation of Non-Woven Materials Made of Poly(ε-Caprolactone) Nanofibers Prepared by Electrospinning under Different Conditions. *J Biomater Sci Polym Ed* **2005**, *16* (12), 1537–1555. https://doi.org/10.1163/156856205774576655.

(11) Li, Y.; Han, C.; Zhang, X.; Bian, J.; Han, L. Rheology, Mechanical Properties, and Biodegradation of Poly(ε- Caprolactone)/Silica Nanocomposites. *Polym Compos* **2013**, *34* (10), 1620–1628. https://doi.org/10.1002/pc.22562.

(12) Allo, B. A.; Rizkalla, A. S.; Mequanint, K. Hydroxyapatite Formation on Sol-Gel Derived Poly(ε-Caprolactone)/ Bioactive Glass Hybrid Biomaterials. *ACS Appl Mater Interfaces* **2012**, *4* (6), 3148–3156. https://doi.org/10.1021/am300487c.

(13) Tallia, F.; Ting, H. K.; Page, S. J.; Clark, J. P.; Li, S.; Sang, T.; Russo, L.; Stevens, M. M.; Hanna, J. V.; Jones, J. R. Bioactive, Degradable and Tough Hybrids through Calcium and Phosphate Incorporation. *Front Mater* **2022**, *9*. https://doi.org/10.3389/fmats.2022.901196.

(14) Mondal, D.; Rizkalla, A. S.; Mequanint, K. Bioactive Borophosphosilicate-Polycaprolactone Hybrid Biomaterials: Via a Non-Aqueous Sol Gel Process. *RSC Adv* **2016**, *6* (95), 92824–92832. https://doi.org/10.1039/c6ra08339k.

(15) Balasubramanian, P.; Grünewald, A.; Detsch, R.; Hupa, L.; Jokic, B.; Tallia, F.; Solanki, A. K.; Jones, J. R.; Boccaccini, A. R. Ion Release, Hydroxyapatite Conversion, and Cytotoxicity of Boron-Containing Bioactive Glass Scaffolds. *Int J Appl Glass Sci* **2016**, *7* (2), 206–215. https://doi.org/10.1111/ijag.12206.

(16) Ezhilarasu, H.; Ramalingam, R.; Dhand, C.; Lakshminarayanan, R.; Sadiq, A.; Gandhimathi, C.; Ramakrishna, S.; Bay, B. H.; Venugopal, J. R.; Srinivasan, D. K. Biocompatible aloe vera and tetracycline hydrochloride loaded hybrid nanofibrous scaffolds for skin tissue engineering. *Int J Mol Sci* **2019**, *20* (20). https://doi.org/10.3390/ijms20205174.

(17) Chen, S. C.; Huang, X. B.; Cai, X. M.; Lu, J.; Yuan, J.; Shen, J. The Influence of Fiber Diameter of Electrospun Poly(Lactic Acid) on Drug Delivery. *Fibers and Polymers* **2012**, *13* (9), 1120–1125. https://doi.org/10.1007/s12221-012-1120-x.

(18) Granel, H.; Bossard, C.; Collignon, A. M.; Wauquier, F.; Lesieur, J.; Rochefort, G. Y.; Jallot, E.; Lao, J.; Wittrant, Y. Bioactive Glass/Polycaprolactone Hybrid with a Dual Cortical/Trabecular Structure for Bone Regeneration. *ACS Appl Bio Mater* **2019**, *2* (8), 3473–3483. https://doi.org/10.1021/acsabm.9b00407.

(19) Arcos, D.; Vallet-Regí, M. Sol-Gel Silica-Based Biomaterials and Bone Tissue Regeneration. *Acta Biomaterialia*. Elsevier BV 2010, pp 2874–2888. https://doi.org/10.1016/j.actbio.2010.02.012.
